# Efficient assays to quantify the life history traits of algal viruses

**DOI:** 10.1128/aem.01659-23

**Published:** 2023-11-21

**Authors:** Eva J. P. Lievens, Irina V. Agarkova, David D. Dunigan, James L. Van Etten, Lutz Becks

**Affiliations:** 1 Aquatic Ecology and Evolution Group, Limnological Institute, University of Konstanz, Konstanz, Germany; 2 Department of Plant Pathology, University of Nebraska-Lincoln, Lincoln, Nebraska, USA; 3 Nebraska Center for Virology, University of Nebraska-Lincoln, Lincoln, Nebraska, USA; University of Queensland, Brisbane, Queensland, Australia

**Keywords:** lytic virus, phycodnavirus, flow virometry, one-step growth assay, survival assay, inactivation assay, kinetic models

## Abstract

Although viral life history traits—the traits that determine reproductive and survival success—are critical to understanding the population dynamics and impact of viruses, they are understudied compared to molecular traits. This discrepancy is partly due to the challenge of phenotyping viral life histories. We developed improved methods to quantify the life cycles of chloroviruses, which are lytic double-stranded DNA viruses that infect unicellular “chlorella-like” algae. We modified classical one-step growth and survival assays by including flow cytometry and kinetic modeling, and applied these to four chlorovirus strains. Together, the modified assays quantified the full life cycle, including adsorption rate, probability of depolarizing the host cell, probability of releasing progeny virions, time until lysis, burst size, specific infectivity, and mortality rate of infectious virions. We also discovered that biphasic or “tailing” decay can occur in chloroviruses, and measured the fraction of infectious virions that resisted decay. The modified assays are more efficient than existing techniques, quantify more traits, and are applicable to other lytic viruses with quantifiable virions. The modified one-step growth assay is suitable for viruses with mutual exclusion, superinfection exclusion, or superinfection immunity; the modified survival assay can be applied to any lytic virus with quantifiable virions.

**IMPORTANCE:**

Viruses play a crucial role in microbial ecosystems by liberating nutrients and regulating the growth of their hosts. These effects are governed by viral life history traits, i.e., by the traits determining viral reproduction and survival. Understanding these traits is essential to predicting viral effects, but measuring them is generally labor intensive. In this study, we present efficient methods to quantify the full life cycle of lytic viruses. We developed these methods for viruses infecting unicellular *Chlorella* algae but expect them to be applicable to other lytic viruses that can be quantified by flow cytometry. By making viral phenotypes accessible, our methods will support research into the diversity and ecological effects of microbial viruses.

## INTRODUCTION

The biology of viruses can be described at various functional levels, from the genetic code to overall fitness. At the molecular levels, next-generation methods have provided detailed information about viral genomes, transcriptomes, and proteomes [e.g., references (1–3)]. For example, metagenomic approaches have started to uncover the extensive genetic diversity of viruses in the world’s virome (4). Life history traits, which quantify the steps in the life cycle, are at a higher functional level. Viral life histories include traits that determine reproduction, e.g., adsorption rate, lysogeny probability, and progeny number, as well as traits that determine survival, e.g., virion mortality rate (“performance” phenotypes, [sensu reference (5)]. Reproduction and survival together shape virus’ population dynamics (6), survival in the environment (7–9), and ecosystem impact (10–12). Despite their importance, viral life history traits are often understudied compared to molecular traits (5). A major contributing factor is the difficulty of measuring these microscopic events. Long-established methods such as one-step growth assays or inactivation assays use plaquing techniques to track virus concentrations over time [( e.g., reference (7, 13)], which is typically labor intensive. Alternative methods can require specialist techniques with microscopic resolution [e.g., reference (14)], which can be challenging to implement.

**Fig 1:**
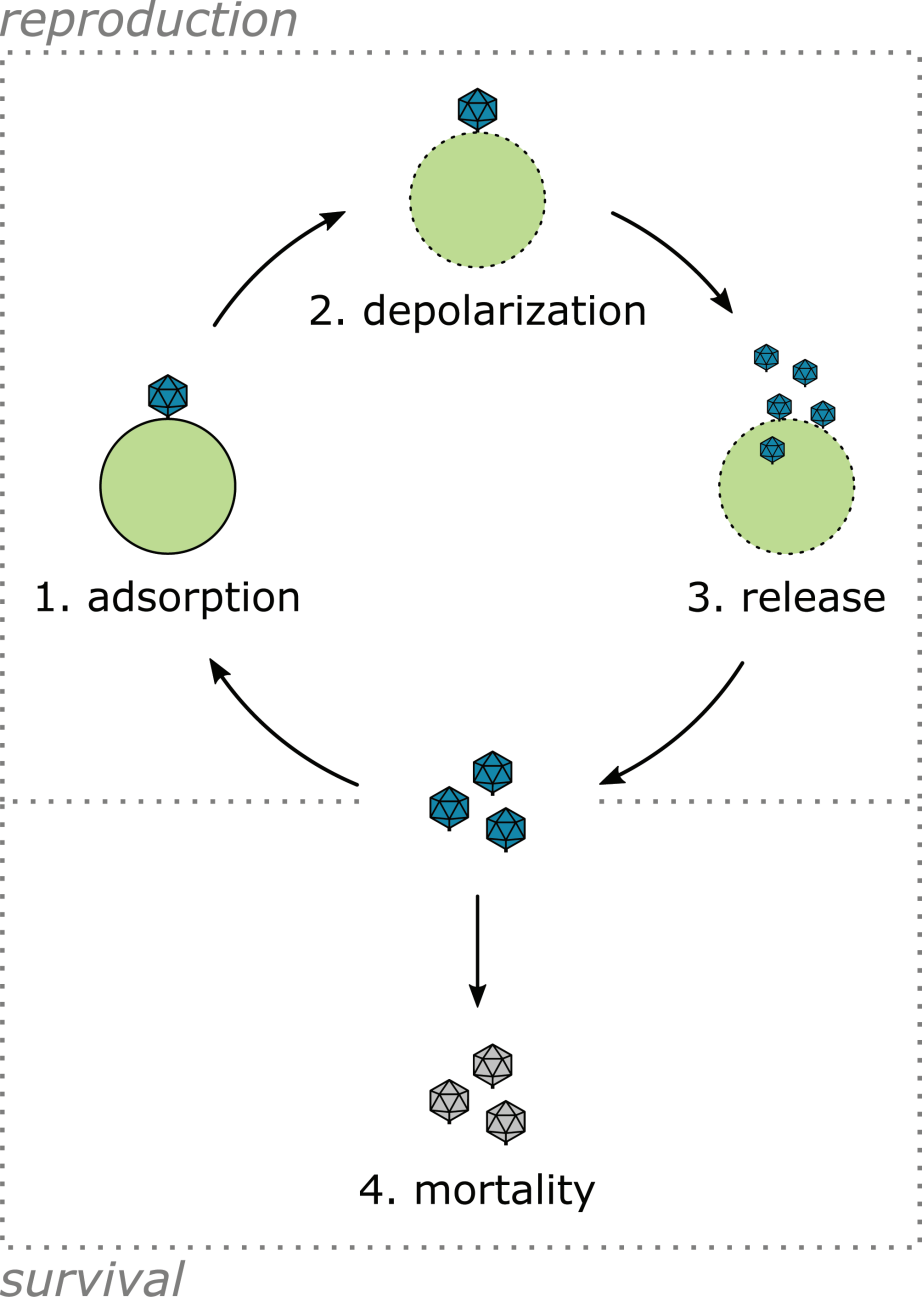
The chlorovirus life cycle can be divided into four quantifiable steps. Only infectious virions are shown. Bursts also include noninfectious virions; for these virions, reproduction fails at steps 1, 2, or 3.

In this study, we present improved methods to measure the life history traits of lytic viruses. We studied the chloroviruses (family Phycodnaviridae, genus *Chlorovirus*), a common aquatic virus model. Chloroviruses are large dsDNA viruses that infect unicellular “chlorella-like” green algae (15). They have a lytic life cycle, which we divide into four quantifiable steps. Steps 1–3 shape the chlorovirus’ reproduction and step 4 its survival (Fig. 1; Table 1). (step 1) Adsorption: Virions adsorb to the host cell wall with the aid of external capsid structures (16); attachment is irreversible without external manipulation (17). (step 2) Depolarization: The adsorbed virion digests a hole in the host cell wall, through which its internal membrane fuses with the host’s plasma membrane (18–20). A virion-associated protein then depolarizes the membrane. Depolarization immediately precedes infection (20) and prevents other chloroviruses from infecting the cell (mutual exclusion) (21). (step 3) Release: After depolarization, viral DNA enters the cell, is replicated in or near the nucleus, and is packaged into ~190 nm capsids in the cytoplasm (19, 22, 23). Mature virions are then released through localized lysis of the plasma membrane and cell wall (24). Virions can be infectious, i.e., capable of catalyzing the death of a host population [forming a plaque or lysing a culture (25)], or noninfectious (not shown in Fig. 1) (18, 25). (step 4) Mortality: Infectious virions decay when exposed to environmental stressors such as light and heat (17, 25).

**TABLE 1:** Traits associated with each life cycle step

Phase	Life cycle step	Associated life history trait
Reproduction	1. Adsorption	Adsorption constant (*k*): determines the rate at which virions adsorb
	2. Depolarization	Depolarization probability (*d*): probability that a virion can depolarize a cell once adsorbed
	3. Release	Lysis time (*μ_l_* ± *σ_l_*): mean ± SD of time until virion release
		Burst size (*b_d_* or *b_r_*): average number of virions produced per depolarized cell (*b_d_*) or per release (*b_r_*)
		Release probability (*r*): probability that a depolarized cell releases infectious virions
	1–3	Specific infectivity (*s*): probability that a virion is infectious, i.e., that it can catalyze the death of a host population
Survival	4. Mortality	Mortality rate (*m*): rate at which infectious virions become noninfectious (only for nonpersistent infectious virions)
		Persistent fraction (*p*): fraction of infectious virions that stay infectious

We developed efficient methods to quantify each step of the chlorovirus life cycle and demonstrate these methods for chlorovirus strains AN69C, CV-K1, KS-1B, and PBCV-1 [strains differing in plaque size and genome size (26)]. A crucial aspect of the assays is the distinction between virions, which we quantified by flow cytometry, and infectious virions, which we quantified based on their ability to lyse host populations. First, a modified one-step growth assay measured traits associated with adsorption, depolarization, and release. Second, a modified survival (mS) assay measured traits associated with virion mortality. Finally, a comparison of the two assays revealed additional insights into progeny release. These methods provide the most thorough quantification of chlorovirus life histories to date and should be applicable to other viruses with similar life cycles.

## RESULTS

### Quantification of reproduction traits (life cycle steps 1–3)

**Fig 2:**
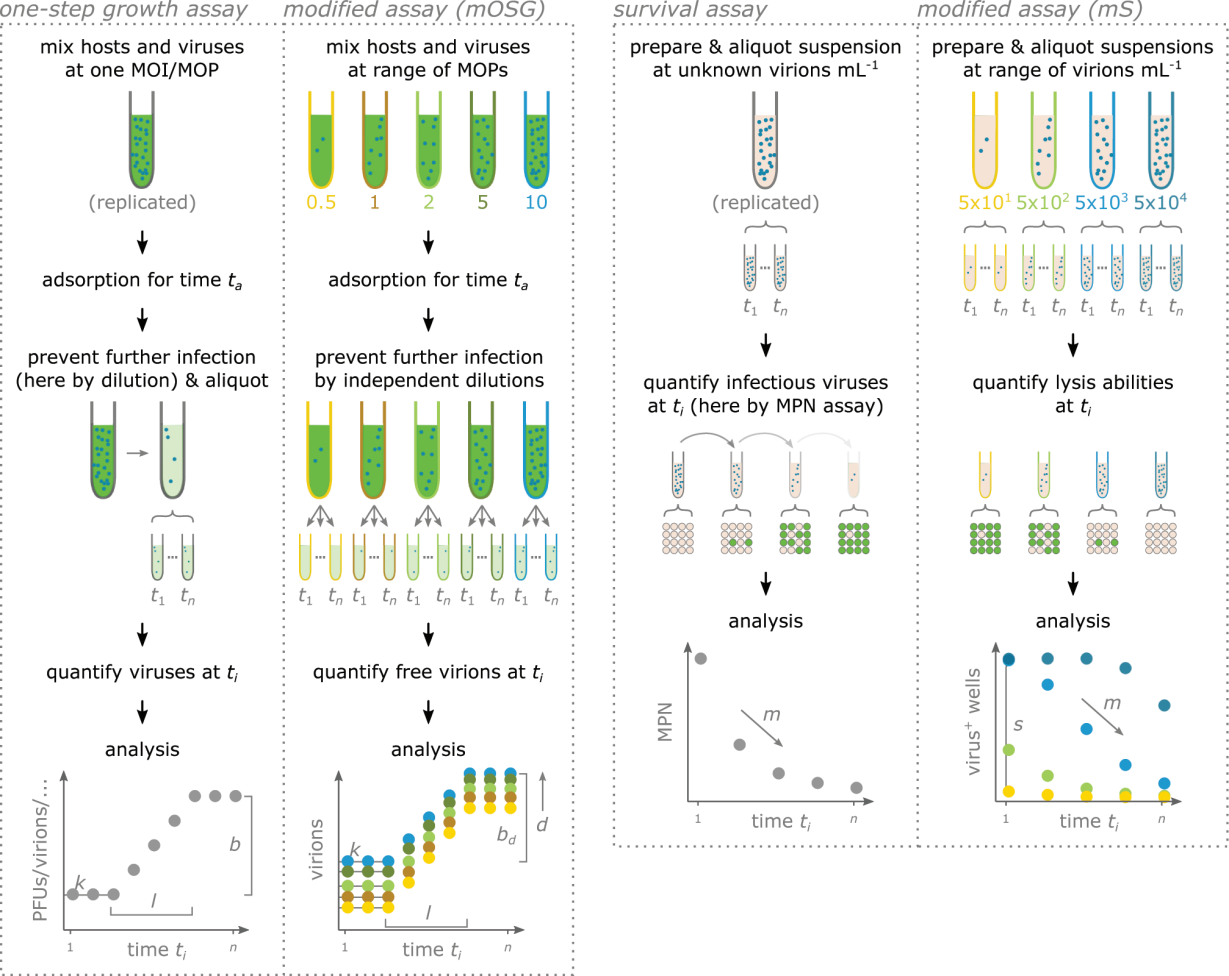
Design of classical vs modified one-step growth and survival assays. See the text for a description. For the classical assays, only one replicate is shown. *b*, burst size; *b*_*d*_, burst size per depolarized cell; *d*, depolarization probability; *k*, adsorption constant; *l*, lysis time; *m*, mortality rate; MPN, most probable number; PFU, plaque-forming unit; *s*, specific infectivity; *t*, time; *t*_*a*_, adsorption time.

**Fig 3:**
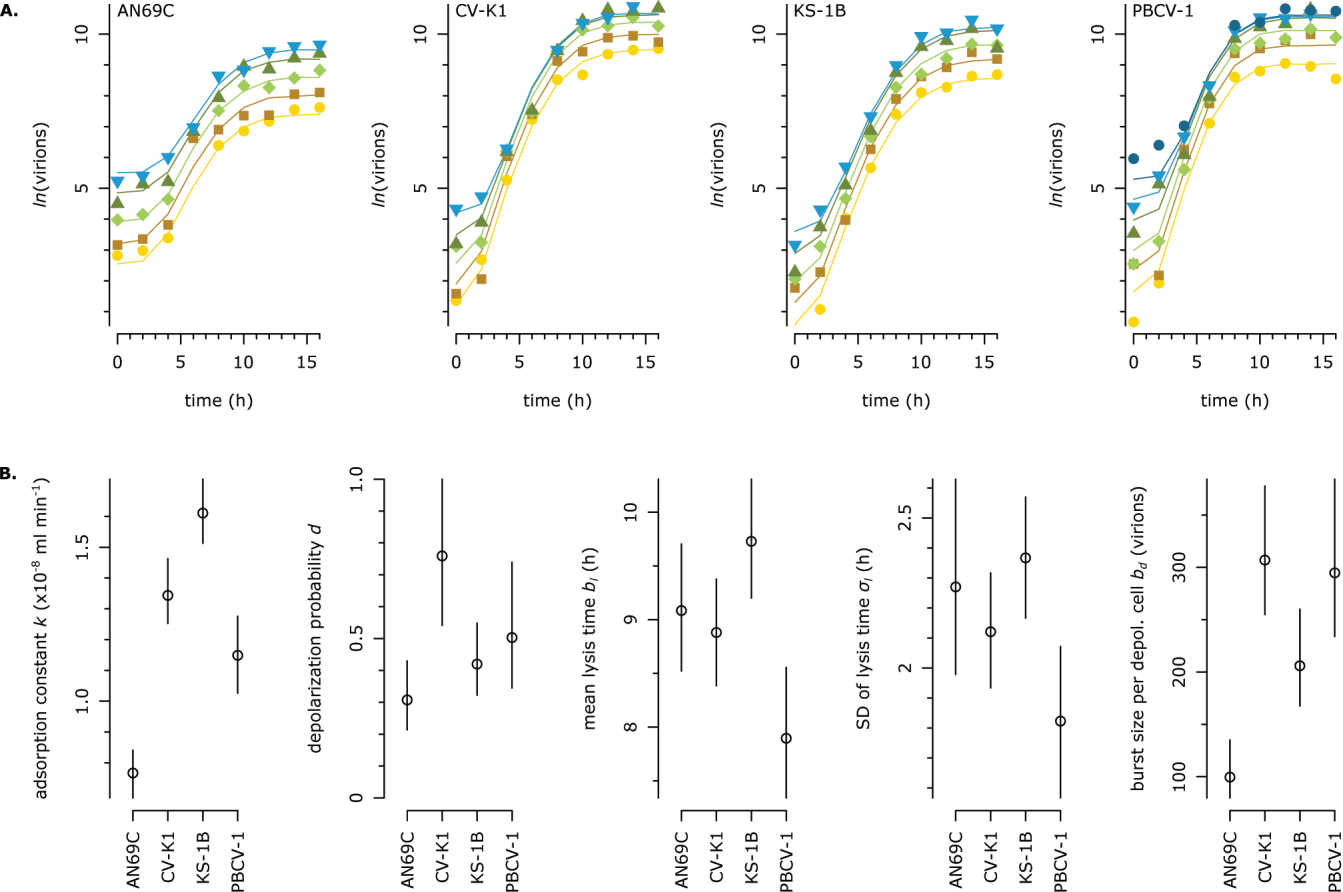
The modified one-step growth assay measures five reproduction traits. (A) Results of the mOSG assay for four virus strains. For each virus strain, we varied the MOP across replicates: MOP 0.5 (yellow circles), 1 (orange squares), 2 (light green diamonds), 5 (dark green triangles), 10 (blue inverted triangles), 20 (indigo circles, only for PBCV-1). The resulting one-step growth curves were measured by flow cytometry, and a kinetic model was fit to the data. Each plot presents the results of one mOSG assay: points represent the observed data (outliers removed); lines represent model predictions. (**B)** Comparison of trait estimates. Bars represent 95% CIs for each trait estimate. For probability traits, axes are scaled to [0, 1].

First, we designed a modified one-step growth (mOSG) assay to quantify the chlorovirus reproduction phase (life cycle steps 1–3, Fig. 1). Our principle modification was to vary the initial virion:host ratios across replicates, i.e., to vary the multiplicity of particles (MOP 0.5, 1, 2, 5, 10, or 20; Fig. 2). This enabled the quantification of an additional trait compared to classical one-step growth assays (see below, Fig. 2). The one-step growth curves were tracked by flow cytometry over 16 hours (points in Fig. 3A), and a kinetic model was fit to the data (lines in Fig. 3A). The model estimated traits for the adsorption, depolarization, and release steps of the life cycle. Adsorption (step 1) was described by the adsorption constant *k*, which determines the rate at which virions adsorb to host cells (27). Depolarization (step 2) was described by the depolarization probability *d*, which we define as the probability that an adsorbed virion is capable of depolarizing a host cell. This trait can be quantified through the variation in MOP. Briefly, as MOP increases, the proportion of depolarized host cells approaches 1. Since depolarization prevents coinfection (21), we can assume that each depolarized host cell produces the same average number of progeny virions (see Discussion for supporting information). Therefore, the total number of progeny virions approaches a maximum as MOP increases, and the depolarization probability can be derived from the rate of this increase. Release (step 3) was described by the mean lysis time across cells *μ_l_*, the standard deviation of lysis time *σ_l_*, and the burst size per depolarized cell *b_d_* (the average number of virions produced by a depolarized cell). The traits are summarized in Table 1, and the trait estimates for AN69C, CV-K1, KS-1B, and PBCV-1 are presented in Fig. 3B.

We confirmed the model’s accuracy by comparing our estimates of the adsorption constant*,* mean lysis time, and burst size per depolarized cell with classical calculations based on the growth curves (Fig. S1). We also performed a separate experiment to measure the mOSG assay’s repeatability and found that it produced consistent estimates of adsorption constant, depolarization probability, mean lysis time, and burst size per depolarized cell (Fig. S2) (28).

### Quantification of survival traits (life cycle step 4) and specific infectivity

**Fig 4:**
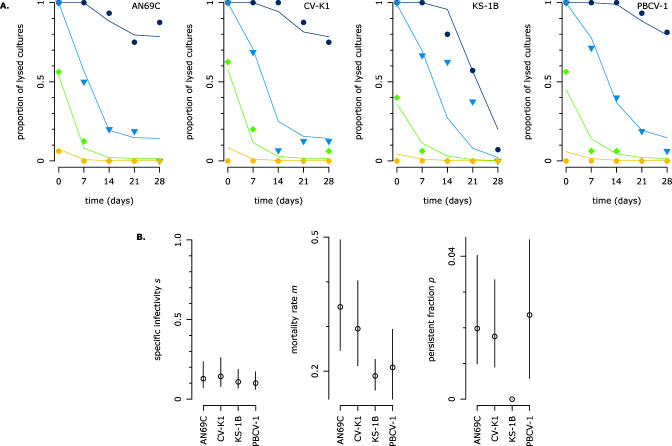
The modified survival assay measures two survival traits and specific infectivity. (A) Results of the mS assay for four virus strains. For each virus strain, we used four initial suspensions: 50 virions mL^−1^ (yellow circles), 500 virions mL^−1^ (light green diamonds), 5,000 virions mL^−1^ (blue inverted triangles), 50,000 virions mL^−1^ (indigo circles). At each time point, an aliquot of each suspension was distributed across 16 liquid cultures of algae. We recorded the proportion of the cultures that lysed within 4 days (i.e., the proportion that contained infectious virions) and fit a kinetic model to this data. Each plot presents the results of one mS assay: points represent the observed data; lines represent model predictions. (**B)** Comparison of trait estimates. Bars represent 95% CIs for each trait estimate. For probability traits, axes are scaled to [0, 1].

We used a modified survival assay to quantify the chlorovirus survival phase (life cycle step 4, Fig. 1). Survival and inactivation assays measure the decline of viral infectiousness after exposure to an environmental stressor, typically using serial dilution methods (Fig. 2). We improved the efficiency of this design by using initial suspensions with known virion concentrations, which we varied across replicates (50, 500, 5,000, or 50,000 virions mL^−1^; Fig. 2). The infectiousness of each suspension was then measured in replicated liquid cultures over the course of 4 weeks (points in Fig. 4A), and a kinetic model was fit to the data (lines in Fig. 4A). We found that mortality was best described by a biphasic model with two classes of infectious virions: “persistent” infectious virions were impervious to decay for the duration of the assay, while “nonpersistent” infectious virions decayed at a constant rate. Thus, chlorovirus mortality (step 4) was described by the persistent fraction *p*, i.e., the proportion of infectious virions that were persistent, and the mortality rate *m* of the nonpersistent infectious virions. Note that we use “persistence” in the environmental sense [cf. (29)] and not as a descriptor of certain types of infections. The model also estimated the specific infectivity *s*, which is the initial proportion of infectious virions. The traits are summarized in Table 1, and the trait estimates for AN69C, CV-K1, KS-1B, and PBCV-1 are presented in Fig. 4B.

We verified the model’s accuracy by comparing our estimates of the specific infectivity*,* mortality rate, and persistent fraction with classical calculations based on the most probable number of infectious virions at each time point (Fig. S1). We also tested the mS assay’s repeatability in a separate experiment, which confirmed the consistency of the estimates (Fig. S3).

### Additional reproduction traits revealed by comparing mOSG and mS assays (life cycle step 3)

**Fig 5:**
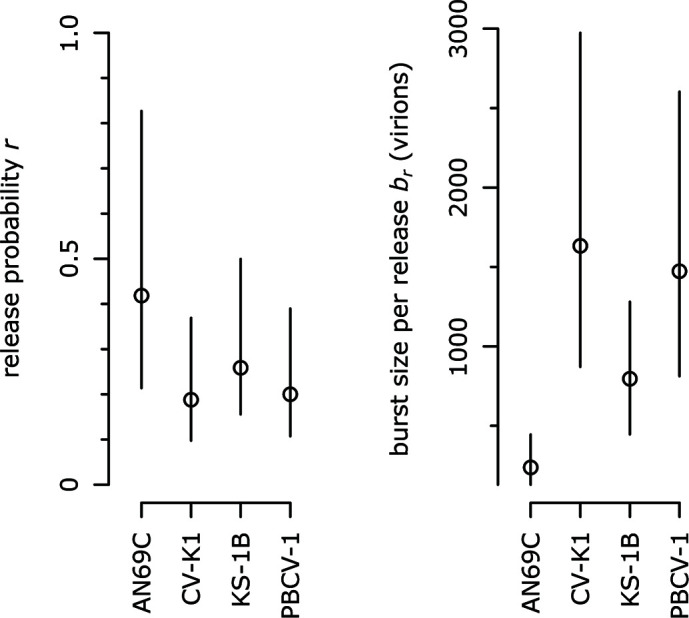
Comparing the modified one-step growth and modified survival assays reveals additional reproduction traits. Comparison of trait estimates. Bars represent 95% CIs for each trait estimate. For probability traits, axes are scaled to [0, 1].

Comparing the results of the mOSG and mS assays allowed us to estimate two final reproduction traits. The specific infectivity *s*, which is the initial proportion of infectious virions in the mS assay, is equivalent to the probability that a virion is capable of adsorbing, depolarizing, and releasing infectious progeny (life cycle steps 1–3, Fig. 1). Assuming that all virions can adsorb if given enough time, this is *s* = *d × r*, where the release probability *r* is the probability that a depolarized cell releases infectious virions. The release probability can then be used to estimate the burst size per release *b_r_* (the average number of virions produced per progeny release event), as *b_d_* = 0 × (1 − *r*) + *b_r_* × *r*. The release probability and burst size per release complete the quantification of the release step in the chlorovirus life cycle (step 3). The additional traits are summarized in Table 1, and the trait estimates for AN69C, CV-K1, KS-1B, and PBCV-1 are presented in Fig. 5.

Independent evidence supporting the calculation of the release probability and burst size per release comes from a single-cell study for a virus related to PBCV-1 [PBCV-1-RK-M2 (30)], which measured a similar burst size per release as estimated here for PBCV-1 (~1,800 and ~1,500 virions, respectively).

## DISCUSSION

### Advantages and applicability of the methods

The mOSG and mS assays represent an important improvement in the measurement of chlorovirus life history traits. By leveraging the distinction between virions and infectious virions, the assays measure more traits than assays based on one or the other [e.g., references (25, 31, 32)]. The estimation of the depolarization and release probabilities is a particular advantage, as these traits are not easily quantifiable using existing methods. (Note that quantifying the release probability does not require the entire mS assay; it can also be obtained by comparing the mOSG assay with a single measurement of specific infectivity.) The modified assays are also more efficient due to the use of high-throughput flow cytometry, strategic manipulation of virion concentrations, and miniaturization into 96-well plates. Finally, the modeling approach maximizes the information gained from each data point and provides bootstrapped 95% CIs for each trait estimate. The latter is particularly important, as the bootstraps can be used to detect and evaluate compensating effects in the model fitting. Compensating effects are inherent correlations between parameters, caused by random noise. For example, a coincidentally lower/higher estimate of virion concentration at time 0 could lead to an over-/underestimate of both adsorption constant and burst size. Such effects might occur in any one-step growth or survival assay; our modeling approach allows them to be detected and compared to the overall trait variation (Fig. S4 and S5).

We expect that these methods will be useful beyond the chloroviruses. The assays are applicable to any culturable lytic virus that can be counted on a flow cytometer, which now includes many aquatic (33) and human viruses (34). The kinetic models can be used as they are or adjusted to reflect life cycle differences. Identical mOSG models could be used for virus species where exclusion is triggered by infection instead of depolarization [superinfection exclusion, e.g., references (35–37)], as well as virus species that coinfect without affecting burst size [superinfection immunity (37)]. In these cases, the model would estimate infection probability instead of depolarization probability and burst size per infected cell instead of burst size per depolarized cell. For virus species where coinfections occur and affect burst size [cf. (32, 38)], the mOSG model could be extended to include a facilitation or depressor effect. The mS model can also be adjusted by including a mortality rate for the persistent fraction [cf. (7, 39, 40)]. Finally, for virus species where the life cycle is less well known, the assays can be used as an explorative tool. An example is shown in Fig. S6: if MOP has no effect on burst size, the relative amplitudes of the mOSG curves should be related to MOP via a Poisson probability function.

### Underlying assumptions

Our approach made several assumptions about chlorovirus biology that could have affected the trait estimates. Although our assumptions were generally well supported, they may not apply under different experimental conditions or to other virus species. Understanding these effects is therefore particularly relevant, and we discuss them in more detail here.

As in the classical one-step growth and survival assays, our methods are predicated on a lytic life cycle. Lysogenic, pseudolysogenic, or budding viruses would not produce the characteristic saturating growth curves of the mOSG assay and might fail to lyse cultures in the mS assay. Thus, prior knowledge of the viral life cycle is required: chloroviruses are not lysogenic and only rarely enter pseudolysogenic states (15).

The kinetic models included two important assumptions. First, we assumed that all virions were capable of adsorbing to host cells. If, in fact, there was a “residual fraction” *f* of non- or slow-adsorbing virions (41), then we would have underestimated the adsorption constant of the “normal” virions. In addition, the specific infectivity would be *s* = (1 − *f*) *× d* × *r*. Thus, our estimates of the release probability would be too low, and consequently our estimates of the burst size per release would be too high. Testing for the presence of a residual fraction could be done by sampling during the adsorption phase of the mOSG assay (27). Second, we assumed that all growth traits were independent of MOP. Higher MOP should not lead to coinfections in chloroviruses, as depolarization causes mutual exclusion (21). We also assume that infections are unaffected by external virions attaching to or digesting the cell wall. Our data were consistent with these assumptions but had limited power to reject it for the traits depolarization probability and burst size per depolarized cell (Fig. S6). External support for MOP independence in these traits comes from a recent single-cell study, which found that burst size per release was unaffected by MOP in a chlorovirus related to PBCV-1 [MOP 1 vs 10 (30)]. However, earlier work did find an effect of multiplicity of infection on burst size in PBCV-1 [MOI 0.1–50 (25)]. It may be that these effects are restricted to very high MOPs/MOIs, when “lysis from without” and coinfection can occur (42, 43). Future work may need to incorporate MOP dependencies, especially at higher MOPs [cf. (32, 38)].

Empirically, we assumed that the mS assay accurately measured the infectiousness of suspensions. Specifically, we assumed that all cultures inoculated with ≥1 infectious virion lysed within 4 days. This assumption is robust for fast-acting chlorovirus strains such as those examined here (E.J.P. Lievens, unpublished data for strain PBCV-1) but may not apply for slow-growing strains. For such strains, it could lead to underestimates of the specific infectivity and, consequently, underestimates of the release probability and overestimates of the burst size per release. To counteract this, the time allowed for cultures to lyse could be extended.

Finally, in order to calculate the release probability and burst size per release, we implicitly assumed that the mOSG and mS assays were comparable. Although the assays were performed under the same conditions, there were some differences. First, the virus suspensions used for the mOSG assay were stored at 4°C for 2–8 days before the assay, while the mS assay suspensions were not. We expect this difference to be negligible, as previous experiments showed that specific infectivity is unaffected by 4°C storage for at least 9 days (J. Clot and E.J.P. Lievens, unpublished data for strain PBCV-1). Second, the host algae were centrifuged before the mOSG assay. This may have affected their susceptibility to depolarization (i.e., *d*) or stability when infected (i.e., *r*), although other experiments do not support such an effect (measured as specific infectivity, i.e., *d × r*; E.J.P. Lievens and M. Spagnuolo, unpublished data for strain PBCV-1). Finally, Covid-19 restrictions imposed a 3-month delay between the two experiments. The environmental differences between these time points, which included separate batches of the host algae, could have affected the specific infectivity. However, this trait is generally robust to such variation (E.J.P. Lievens, unpublished data for various strains).

### New insights: characteristics of defective virions

Defective virions are a well-known aspect of virus biology (44); in chloroviruses, they include empty capsids and capsids with different membrane conformations [strain PBCV-1 (17)]. We found that only 10%–14% of virions were infectious (specific infectivity *s*, Fig. 4). This is lower than what was found in previous work [10% compared to 25%–30% for PBCV-1 (25)], which might be explained by the lower richness of the medium for the algal host [cf. (45–48)], by a difference in sensitivity of liquid culture vs plaque assays, or by a higher concentration of attachment-blocking host receptors in our virus suspensions (18). We were able to quantify two components of infectiousness. Our results suggest that the processes between depolarization and release (DNA entry, replication, packaging, and lysis; 19%–42% success rate, Fig. 5) are more susceptible to error than the processes between adsorption and depolarization (digestion of the cell wall, membrane fusion, and depolarization; 31%–76% success rate, Fig. 3).

The ability to assess whether virus strains are more likely to fail at depolarizing (or infecting) a host cell or releasing infectious progeny could have further applications. Quantifying the proportion of infected vs productive cells can be highly relevant to judge a virus’ impact on a host population—in particular, virus variants with low release probabilities may cause disproportionate amounts of host death for their growth rate. Measuring depolarization and release probabilities can also be a useful way to link omics to phenotypes. For example, future work on the chloroviruses could test whether differences in depolarization probability are linked to differences in efficiency or copy number of the proteins that digest the host cell wall and depolarize the host membrane (digestion: PBCV-1 A561L-homolog, copy number ~60 in PBCV-1, (1, 18) [depolarization: various K^+^ channel proteins (20, 49)].

### New insights: biphasic mortality

Of the four chloroviruses assayed here, three had biphasic or “tailing” mortality. In these strains, 2% of the infectious virions were resistant to decay for the duration of our experiment (persistent fraction *p*, Fig. 4). Biphasic viral decay can be caused by several factors: (i) Changes in medium properties through time (7). In this case, we would have expected the same biphasic pattern to occur for all four strains, so this is unlikely. (ii) Adhesion to organic particles (17, 29). In this case, we would expect to see a larger persistent fraction in faster-growing strains because they cause more lysis and thus produce more organic matter; we do not see this pattern (data not shown). (iii) Viral aggregation (29). (iv) Two concurrent mortality processes causing differing degrees of damage [cf. (7, 50–52)]. The first process (e.g., damage to the capsid) would need to occur rapidly but leaves some probability of successful infection, while the second process (e.g., DNA denaturation) occurs slowly and leads to complete inactivation. (v) The presence of a more resistant subpopulation with slower decay. These might be virions with fewer production errors (40) or virions with a specifically different phenotype (7, 39). We expect mechanism 3, 4, or 5 to be acting on the chloroviruses; further work will be necessary to distinguish between them and to understand why the persistent fraction varies across strains.

It is striking that we found biphasic decay because the existing work on phytoplankton viruses typically assumes decay is exponential [8, 9, i.e., mortality at a constant rate; (8, 9, 53–58)]. Though assuming exponential decay may be appropriate in many cases, it seems unlikely that the chloroviruses are the only phytoplankton viruses with a persistent fraction [ e.g., possible nonlinearity in reference (8, 31)]. Investigating persistence in other phytoplankton viruses could resolve some questions about their ecology, such as their ability to form “seed banks” that persist outside blooms (8). It would also affect our understanding of aquatic microbial communities and nutrient cycling, in which viral infection and decay play important roles (59).

### Conclusion

The ability to phenotype viral life histories is essential to understanding viral fitness, epidemiology, and impact on the host population (6, 9, 41). Due to their efficiency, the methods developed in this study are particularly useful to compare viruses—whether across virus strains, across environmental conditions, or even across variants within strains [e.g., virion types (17)]. The assays will be used to explore the diversity and evolution of chloroviruses in future, and we expect them to provide similar insights into other lytic viruses with quantifiable virions.

## MATERIALS AND METHODS

### Materials and experimental conditions

Unless otherwise specified, assays took place at 20°C and under constant light. Algal growth and virus incubations were done on an orbital shaker with diameter 10 mm and frequency 120 rpm. Any storage at 4°C was also dark storage. “Culture plates” are always 96-well flat-bottomed tissue culture plates (Techno Plastic Products, CH); “deep well plates” are always 96-well 2.2-mL polypropylene deep well plates (VWR International, USA).

We demonstrated the methods for four chlorovirus strains: AN69C, CV-K1 (also called CviKI), KS-1B, and PBCV-1. Virus suspensions were stored in lysate form at 4°C. Before the assays, we refreshed these suspensions as follows: 0.5 mL of the lysate was inoculated into 10 mL of 2  ×  10^6^ algae/mL suspension, incubated for 24 h, and filtered through 0.2 µm. The filtrates were stored at 4°C, and their virion concentrations were measured by flow cytometry (see below). The filtrates with insufficient virion concentrations were given one more round of this amplification treatment.

As a host, we used the unicellular green alga *Chlorella variabilis* NC64A. The alga was stored on agar slants at 4°C and inoculated into liquid medium before use. We used a modified version of Bold’s Basal Medium (BBM) (60), with ammonium chloride substituted for sodium nitrate as a nitrogen source and double the concentration of trace element solution 4 [first used by reference (61)]. We kept the abbreviation BBM in order to distinguish our medium from the enriched “MBBM” typically used in this model system [e.g., reference (25)]. Algae used in the assays were in late exponential phase (~2  ×  10^6^ algae/mL in this medium).

### Virion quantification with flow cytometry

To quantify virions, we used a flow cytometry protocol based on Brussaard (33) and Wen et al. (62). The protocol does not require fixation and can handle up to 96 samples at a time with 97%–99% repeatability (data not shown). Briefly, virus samples were mixed with SYBR Green I stain (Merck, Germany) and Tris-EDTA buffer in a PCR plate, to a final volume of 150 µL and a final SYBR Green concentration of 0.5×. Depending on the expected virion concentration, 7.5 or 30 µL of virus sample was used. The samples were heated to 80°C for 10 min, allowed to cool for 15 min, and stained particles were counted by a flow cytometer with automated loader (BD FACSVerse, BD Biosciences, USA). Each sample was run for 30 s (40–50 μL), and the virion population was identified based on its side scatter and green fluorescence. As DNA staining was necessary to identify virions, we expect this population to exclude empty capsids (17). A detailed protocol is available on protocols.io (63).

### Modified one-step growth assay

**Fig 6:**
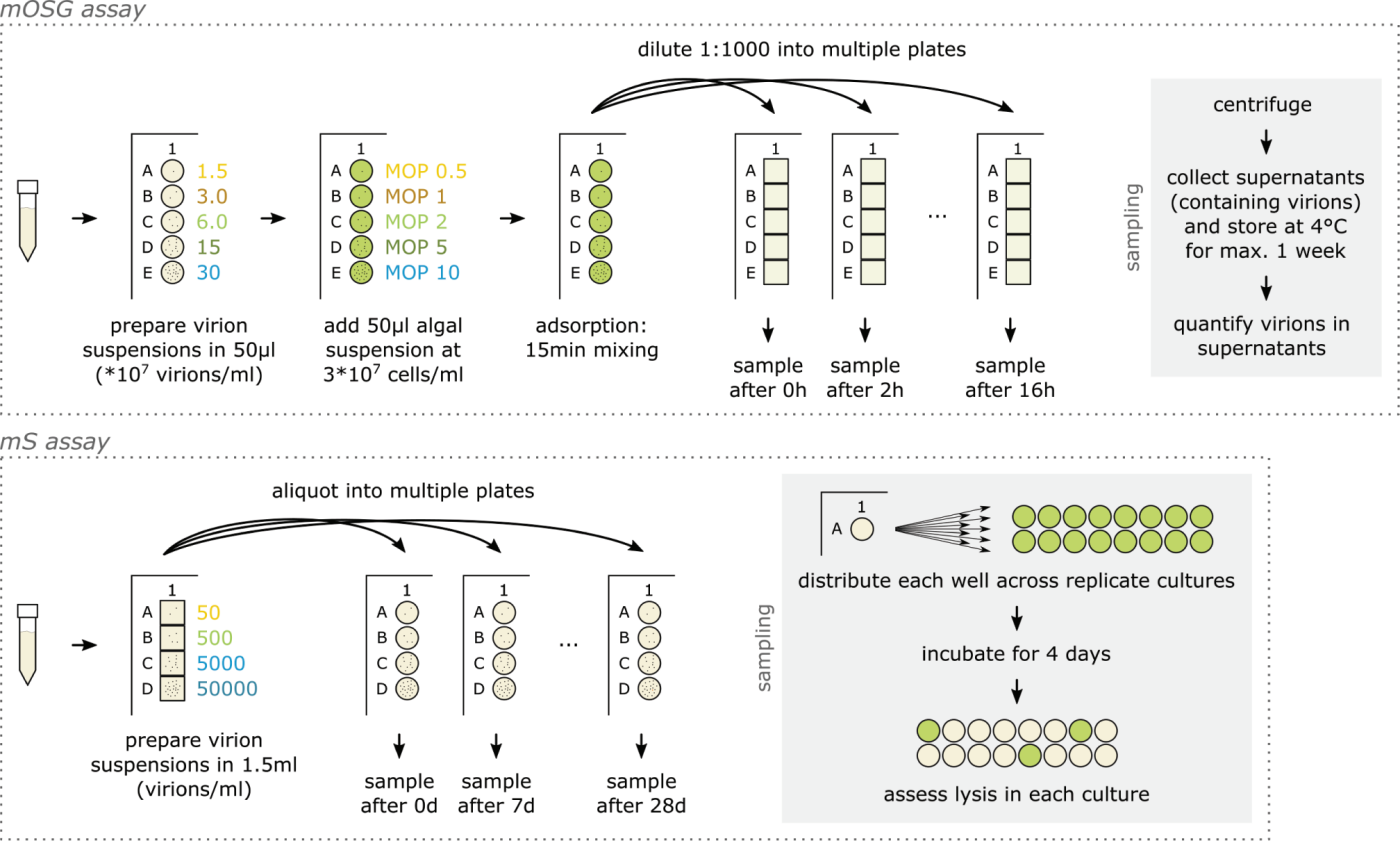
Schematic representation of the mOSG and mS assays for one virus strain. Filtrates are represented by 15 mL tubes; culture plates are depicted with round wells; deep well plates are depicted with square wells. Solid gray boxes describe the sampling procedure that was repeated at every time point.

Our modifications to the classical one-step growth assay were (i) varying the MOP across replicates [cf. (32, 38)], (ii) improving throughput by miniaturizing the assay into 96-well plates, and (iii) improving throughput by using flow cytometry to measure the output [cf. (38)]. The mOSG assay was performed as follows. We combined algae and viruses in culture plates; each well contained 0.1 mL suspension with 1.5  ×  10^7^ algae/mL and either MOP 0.5, 1, 2, 5, or 10 of a given virus filtrate (MOP 20 was included for strain PBCV-1). The virus volumes were pipetted separately, so that the different MOPs were statistically independent. Immediately after combining algae and viruses, the suspensions were mixed for 15 min to induce adsorption (1,200 rpm on a microplate shaker with orbital diameter 1 mm). The infections were then synchronized by 1,000-fold dilutions into nine sets of deep well plates (2 µL suspension + 1,998 µL BBM). Each dilution was pipetted separately to maximize the statistical independence of the data. The deep well plates were sealed with transparent foil and left standing; they were inverted every hour to prevent sedimentation. At time points 0, 2, 4, …, and 16 h after dilution, we sampled a set of deep well plates: deep well plates were centrifuged for 15 min at 2,000 g to separate virions from algae, after which supernatants (containing free virions) were transferred into culture plates and stored at 4°C. At the time of dilution, we also prepared one deep well plate containing a 1:10,000 dilution. It was sampled after 16 h and served to detect signals of secondary infection (see Fig. S7M). The virion concentration in the supernatants was quantified by flow cytometry within 1 week of sampling. The protocol is represented schematically for one virus strain in Fig. 6; a detailed protocol is available on protocols.io (64).

The mOSG assay produced five growth curves per virus strain (six for strain PBCV-1). Each curve included virions that had not adsorbed during the adsorption period and newly released progeny virions. We modeled the curves as


(Eq. 1) $$V\left(t\right)=\frac{1}{\delta }\times \left(\underset{unadsorbed\;virions}{\underbrace{A_{a}\times M\times e^{-k\times A_{a}\times t_{a}}}}+\underset{release\;of\;progeny\;virions}{\underbrace{A_{a}\times \left(1-e^{-d\times \left(1-e^{-k\times A_{a}\times t_{a}}\right)\times M}\right)\times F\left(t;\mu_{l},\sigma_{l}^{2},\alpha \right)\times b_{d}}}\right)$$

Here, *V(t*) is the free virion concentration at *t* hours after dilution, *M* is the MOP (0.5–20), and *δ* is the dilution factor (1,000). Assuming that virions adsorbed at a constant rate, that all virions were capable of adsorbing, and that there were enough binding sites available for all virions (65), the concentration of unadsorbed virions at the end of the adsorption period follows an exponential model with adsorption constant *k*, algal concentration during the adsorption period *A_a_* (1.5  ×  10^7^ algae/mL), duration of the adsorption period in minutes *t_a_* (15 min), and initial virion concentration *A_a_ × M* (27, equation 18.2). The concentration of progeny virions over time depends on the concentration of depolarized cells, the time at which they lyse, and their burst size upon lysis. The concentration of depolarized cells at the end of the adsorption period is derived from the Poisson distribution with expected number of events $d\times \left(1-e^{-k\times A_{a}\times t_{a}}\right)\times M$, which is the probability that an adsorbed virion is capable of depolarization *d* multiplied by the multiplicity of adsorption [sensu reference (27)]. As discussed above, we assumed that MOP had no effect on the average number of progeny virions produced per depolarized cell *b_d_*. Under the simplification that all depolarized host cells produced *b_d_* virions, the proportion of lysed cells over time *F(t*) could be approximated by the cumulative distribution function of a truncated normal distribution with mean lysis time *μ_l_*, standard deviation *σ_l_*, and the earliest possible lysis time α [in reality, the burst size probably varied across cells (30, 66, 67), but without single-cell information we could not take this into account]. We set α to 0 for simplicity, as including α as a separate parameter had minimal effects on the fit (Fig. S8). The model assumed that free virions were not lost within the time frame of the experiment (e.g., by mortality or adsorption to debris); this was supported by the flat shape of the growth curves at the last time points. Equation 1 is derived in more detail in Method S1, and the parameters are summarized in Table 1. We investigated the model’s sensitivity by varying each parameter and evaluating its effect on a simulated data set; the effects are presented graphically in Fig. S9.

For each viral strain, we fit equation 1 to our virion concentration data using nonlinear least squares fitting [“nls” in base R version 3.6.1 (68)]. Function *F(t*) was calculated with the “EnvStats” package (69). The data were *ln* transformed and curated before fitting: obvious outliers were removed (virion concentration implausibly high or low compared to the data for other MOPs at the same time point, or compared to other time points of that MOP), as were time points with secondary infections (aberrant 1:1,000 vs 1:10,000 dilutions data at time point 16 h, or secondary increases at later time points for low MOPs). For the fitting, all parameters were given appropriate lower bounds (0 for *k* and *d*; 0.5 for *σ_l_*; 1 for *μ_l_* and *b_d_*). *k*, *σ_l_, μ_l_*, and *b_d_* were given unrealistically high upper bounds; *d* was given a meaningful upper bound of 1. To ensure the parameter estimates were robust, we used a variety of initial parameters for each model (example in Fig. S7, panels A–D). The quality of the model fit was assessed by visually judging the fit and distribution of residuals (example in Fig. S7, panels I–O). Nonparametric bootstrapping was used to obtain 95% CI*s* for each parameter [package “nlstools,” (70)] (example in Fig. S7, panels E–H).

### Modified survival assay

Before the mS assay, we produced “fresh” viruses by amplifying viruses in 10 mL of 1  ×  10^6^ algae/mL suspension. The amplification was started with a modest number of virions from the filtrates (~5  ×  10^5^ virions/mL) and incubated for 24 h. Suspensions were then filtered through 0.45 µm, and the virion concentration in the filtrates was immediately quantified by flow cytometry.

Our modifications to the typical survival or inactivation assays [e.g., references (53, 71)] were (i) using virus suspensions with known virion concentrations, (ii) improving throughput by varying the initial virion concentration across replicates (instead of serially diluting at each time point), and (iii) improving throughput by miniaturizing the assay into 96-well plates. The mS assay was run on the refreshed filtrates as follows. In deep well plates, we combined filtrates and BBM to produce 1.5 mL suspensions containing 5  ×  10^4^, 5  ×  10^3^, 5  ×  10^2^, and 5  ×  10^1^ virions/mL of a given virus strain. The filtrate volumes were pipetted separately, so that the different suspensions were statistically independent. The suspensions were mixed and aliquoted into five sets of culture plates, which were sealed with transparent foil and left standing. After 0, 7, 14, 21, and 28 days, we used a set of culture plates to measure the concentration of infectious virions in each suspension: 10 µL from each suspension was added to 16 wells containing 190 µL of 1.05  ×  10^6^ algae/mL suspension (also in culture plates). These were incubated for 4 days, after which we measured the OD_680_ to determine which culture wells had lysed. The protocol is represented schematically for one virus strain in Fig. 6; a detailed protocol is available on protocols.io (72).

Over time, the proportion of lysed wells decreased. We modeled this decline as


(Eq. 2) $$P\left(t\right)=1-e^{-\left(V\times s\times p+V\times s\times \left(1-p\right)\times e^{-m\times t}\right)}$$

This biphasic decay model assumes that infectious virions can be “nonpersistent,” i.e., susceptible to decay, or “persistent,” i.e., immortal for the duration of our assay. We used this model because the exponential decay model was often a poor fit (Fig. S10). This biphasic parametrization was the simplest extension of the exponential model and fit our data very well. *P(t*) is the proportion of lysed wells after *t* days, and $V\times s\times p+V\times s\times \left(1-p\right)\times e^{-m\times t}$ is the expected number of infectious virions added to each culture well. *V* is the initial number of virions (e.g., 10 µL of a 5  ×  10^4^ virions/mL suspension =  500 virions), *s* is the frequency of infectious virions when the suspension is fresh, *p* is the fraction of those virions that is persistent, and *m* is the mortality rate of the nonpersistent infectious virions (fraction 1 − *p*). The probability that at least one infectious virion was added to a well is derived from the Poisson distribution. Equation 2 is derived in more detail in Method S1, and the parameters are summarized in Table 1. We investigated the model’s sensitivity by varying each parameter and evaluating its effect on a simulated data set; the effects are presented graphically in Fig. S11.

For each viral strain, we fit equation 2 to the data using generalized nonlinear regression models, with the number of lysed vs unlysed wells as a binomial response variable [in R version 3.6.1 (68), package “gnlm” (73)]. The parameters *s*, *p*, and *m* were inverse logit transformed to keep them in the interval [0,1] (*m* can be larger than 1, but in practice, the mortality rates were much lower). To ensure the parameter estimates were robust, we used a variety of initial parameters for each model (example in Fig. S12, panels A–C). The quality of the model fit was assessed by visually judging the fit and distribution of residuals (example in Fig. S12, panels G–L). Parametric bootstrapping was used to obtain 95% CIs for each parameter (example in Fig. S12, panels D–F).

### Comparison of mOSG and mS assays

The release probability *r* was derived from the specific infectivity *s* and the depolarization probability *d* (see above or Method S1):


(Eq. 3) r=s/d

We then derived the average burst size per release *b_r_* as


(Eq. 4) $$b_{r}=b_{d}/r$$

because the burst size of depolarized cells that do not release infectious virions is 0 (see above or Method S1). Note the implicit assumption that all bursts contain a mix of infectious and noninfectious virions (as opposed to some depolarized cells producing only noninfectious virions). The bootstraps from the mOSG and mS assays were used to calculate 95% CIs for *r* and *b_r_*.

## Data Availability

Data and modeling code is available at doi:10.5281/zenodo.6573770.

## SUPPLEMENTAL MATERIAL

Supplemental file 1 aem.01659-23-s0001.pdf Fig. S1 to S12.

Supplemental file 2 aem.01659-23-s0002.pdf Supplemental methods.

ASM does not own the copyrights to Supplemental Material that may be linked to, or accessed through, an article. The authors have granted ASM a non-exclusive, world-wide license to publish the Supplemental Material files. Please contact the corresponding author directly for reuse.
