# Supplementary material for: Efficient assays to quantify the life history traits of algal viruses
Source: Appl Environ Microbiol. 2023 Nov 21;89(12):e01659-23. doi: 10.1128/aem.01659-23 (PMC10734466; doi:10.1128/aem.01659-23)
Supplement: Supplemental file 1 — Fig. S1 to S12. [file aem.01659-23-s0001.pdf]

Notes on the supplemental figures:

The supplemental figures are numbered according to their appearance in the text. **From a methodological perspective, the following order is most informative:**

1. Methods development
   - Development of the kinetic models: S9 (mOSG assay) & S11 (mS assay)
   - Fitting the kinetic models to the data: S7 (mOSG assay) & S12 (mS assay)
   - Exploration of biological assumptions: S6 (mOSG assay)
   - Reliability of the methods: S1 (both assays), S2 (mOSG assay), S3 (mS assay)
2. Methods application
   - Model simplifications: S8* (mOSG assay), S10* (mS assay)
3. Interpretation of results
   - Impact of correlations between model parameters: S4 & S5 (both assays)

***AN69C, CV-K1, KS-1B, and PBCV-1 were phenotyped as part of a larger chlorovirus dataset.** We reference the larger chlorovirus dataset when presenting the simplifications of the kinetic models, because these should be chosen in light of the entire dataset (e.g. in Lievens et al. 2022, α could not be set to 0).

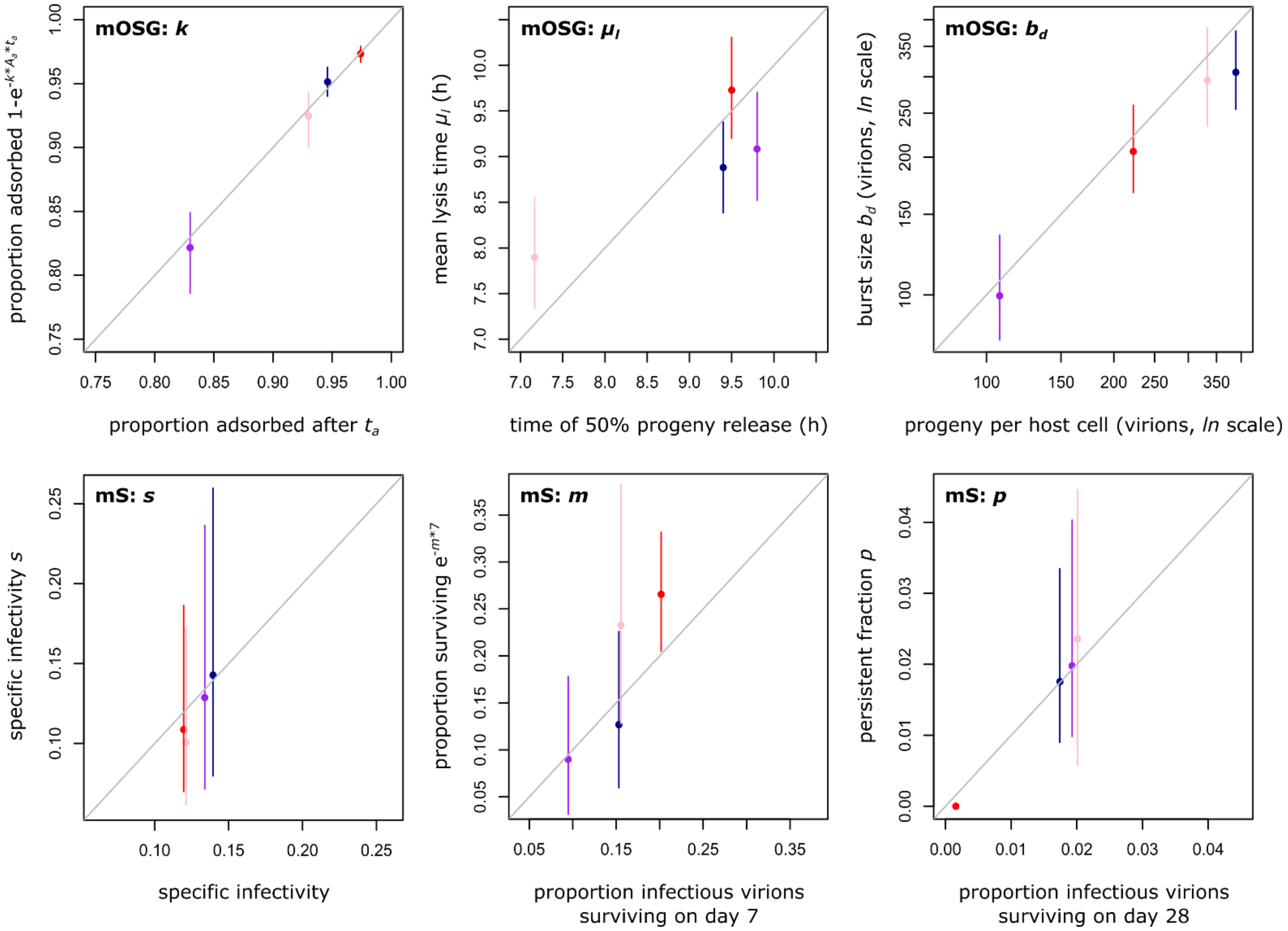

Supp. Fig. S1. Comparing the trait estimates from Eqs. 1 & 2 to 'classical' calculations. Each plot shows a trait quantified by the mOSG or mS assay (purple: AN69C, blue: CV-K1, red: KS-1B, pink: PBCV-1). The x-axes represent 'classical' calculations, which are based purely on the curated data: ***k*)** the proportion of adsorbed virions at time 0[1], calculated for each MOP and averaged; ***μ_l*)** the time at which the growth curve reached half of its amplitude[2] (specifically the midpoint of the 2 h interval in which this occurred), calculated for each MOP and averaged; ***b_d*)** the amplitude of the growth curve[2] divided by the total number of host cells, calculated for MOPs ≥ 9 and averaged; ***s*)** the most probable number[3] (MPN) of infectious virions on day 0 divided by the concentration of virions added; ***m*)** the MPN of infectious virions on day 7 divided by the MPN of infectious virions on day 0; ***p*)** the MPN of infectious virions on day 28 divided by the MPN of infectious virions on day 0. The y-axes show a comparable phenotype based on the retained trait estimates. Vertical bars represent the 95 % CIs of the retained trait estimates; the gray line is the 1 : 1 line.

---

[1] proportion of adsorbed virions at time 0 = $1 - \frac{V(0)}{(A_a * M * {}^{1}/_{\delta})}$

[2] amplitude = $\frac{V(2nd-to-last\ time\ point)+V(last\ time\ point)}{2} - V(0)$

[3] MPN calculated from the number of virus-positive and virus-negative wells using the R package 'MPN' (Ferguson & Ihrie, https://cran.r-project.org/web/packages/MPN/index.html)

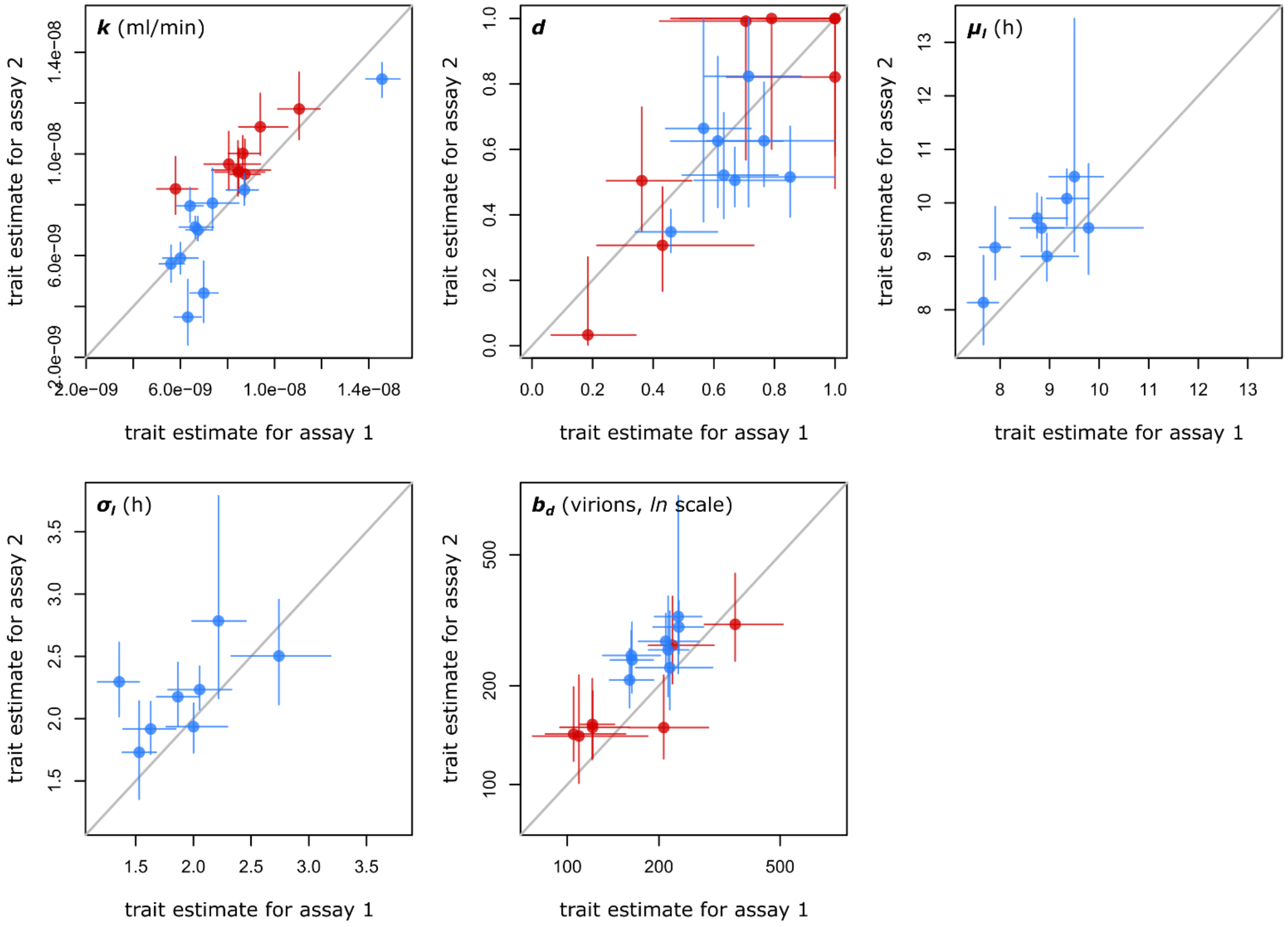

Supp. Fig. S2. Repeatability of the mOSG assay. Different viruses were subjected to two mOSG assays within the span of a week. The assays used four MOPs (0.5, 1, 5, 10) and time points up to 14 h. Eq. 1 was fit to the data from each day: the point represents the trait estimate, and the lines represent the 95 % CIs. Various chlorovirus strains are shown in blue, while PBCV-1 genotypes isolated from Retel, Kowallik et al. (2019, E.J.P. Lievens unpublished data) are shown in red. The latter group were not included in the “no effect of testing day” analyses of *k* due to inconsistency in the shaker; they are not included in the analyses of μ_l and σ_l due to MOP-dependencies in lysis time (Lievens et al. 2022). Incomplete assays and CIs that were too broad to be informative were removed. The gray line is the 1 : 1 line.

We quantified repeatability as the proportion of phenotypic variation that could be attributed to between-strain variation (Nakagawa & Schielzeth 2010). Repeatability ranges from 0 to 1; higher repeatabilities imply lower measurement error and/or higher variation between strains. The repeatabilities of the trait estimates were 0.86 for the adsorption constant *k*, 0.82 for the depolarization probability *d*, 0.51 for the mean lysis time μ_l, 0.44 for the SD of lysis time σ_l, and 0.65 for the burst size per depolarized cell b_d. When an effect of the testing day was included to account for environmental variation, repeatability increased to 0.76 for the mean lysis time μ_l, 0.57 for the SD of lysis time σ_l, and 0.83 for the burst size per depolarized cell b_d. Note that we used more MOPs, more time points, and a single testing day in the mOSG assays presented in the main text, so our power was certainly higher.

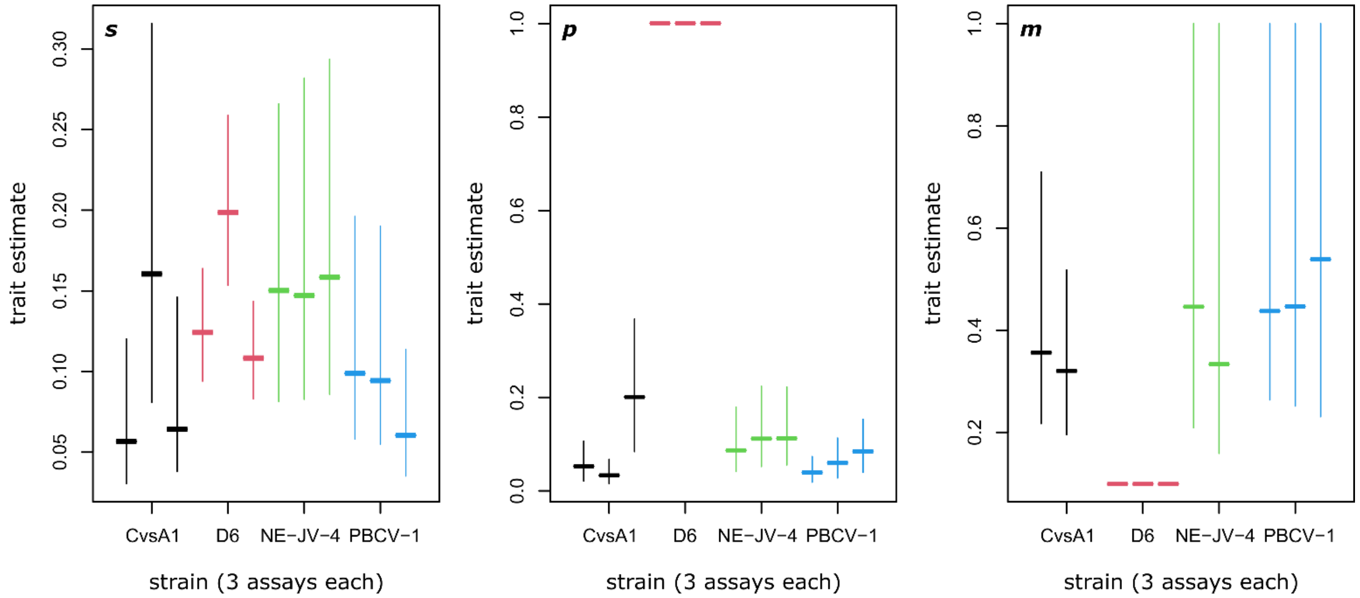

Supp. Fig. S3. Repeatability of the mS assay. Four different viruses were subjected to three mS assays in parallel, with sampling every 8 days for 32 days. Eq. 2 was fit to the data from each replicate: the horizontal bar represents the trait estimate, and the vertical lines represent the 95 % CIs. Replicates of strain CvsA1 are shown in black; of PBCV-1-RK-D6 (isolated from Retel et al. 2019, E.J.P. Lievens unpublished data) in red; of strain NE-JV-4 in green; of the original PBCV-1 strain in blue. CIs that were too broad were removed according to the same criteria as above. Data from Lievens et al. *in prep.*

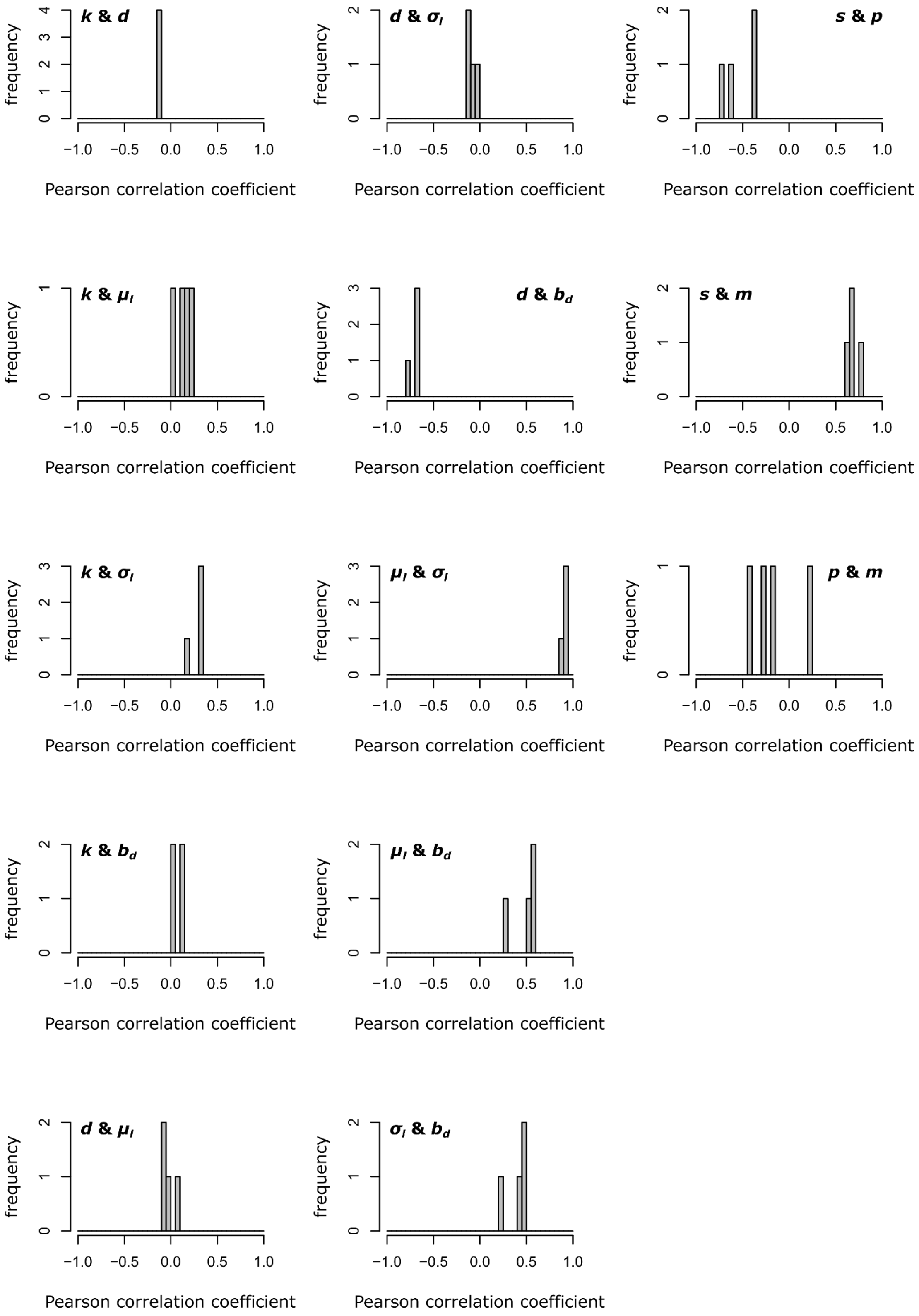

Supp. Fig. S4. Correlations between model parameters arising from the fitting process. Because the mOSG and mS datasets are finite, it is possible that some correlations between model parameters arise purely from the fitting process. For example, when *s* and *m* are fitted to highly similar datasets, the fitted *m* will be higher if the fitted *s* is higher. To judge whether this occurs, we used the highly similar datasets generated by bootstrapping the 95 % CIs (see Supp. Figs. S9 and S12). We calculated the Pearson correlation coefficient of the bootstrap values for every parameter combination for every viral strain. The histograms of these correlation coefficients are plotted here, with one parameter combination per panel. Consistent correlations occurred between: d & b_d, μ_l & σ_l, μ_l & b_d, σ_l & b_l, s & p, and s & m. Interpretation example: the bootstrap values of *p* and *m* (bottom right) were negatively correlated for 3 virus strains, and positively correlated for 1 virus strain.

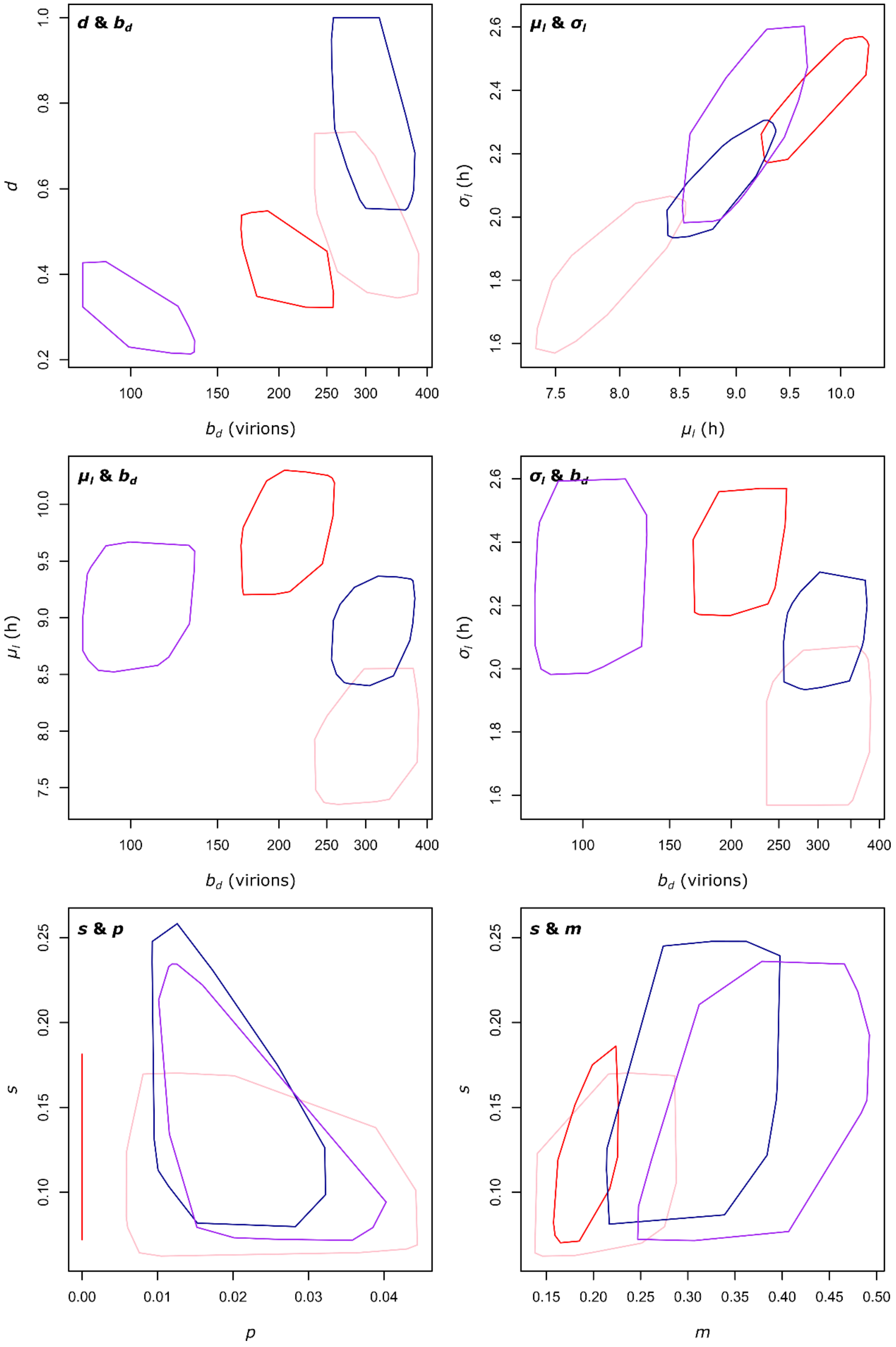

Supp. Fig. S5. Effect of correlations between model parameters arising from the fitting process. Before interpreting the trait estimates, it is important to look for confounding effects of the parameter correlations described in Supp. Fig. S4. To do so, the CIs of the correlated parameter combinations (d & b_d, μ_l & σ_l, μ_l & b_d, σ_l & b_l, s & p, and s & m) should be compared to the overall range of those parameter combinations (i.e. to the phenotype space). Each panel shows a parameter combination, and each polygon represents one viral strain (purple: AN69C, blue: CV-K1, red: KS-1B, pink: PBCV-1). Polygons outline the bootstrap values that fell within the 95 % CIs for a given parameter combination. For e.g. d and b_d, there is little overlap between polygons, so parameter correlations would not affect the interpretation of the relative trait estimates. For e.g. s and p, the overlapping polygons indicate that correlations caused by model fitting cannot be distinguished from phenotypic correlations.

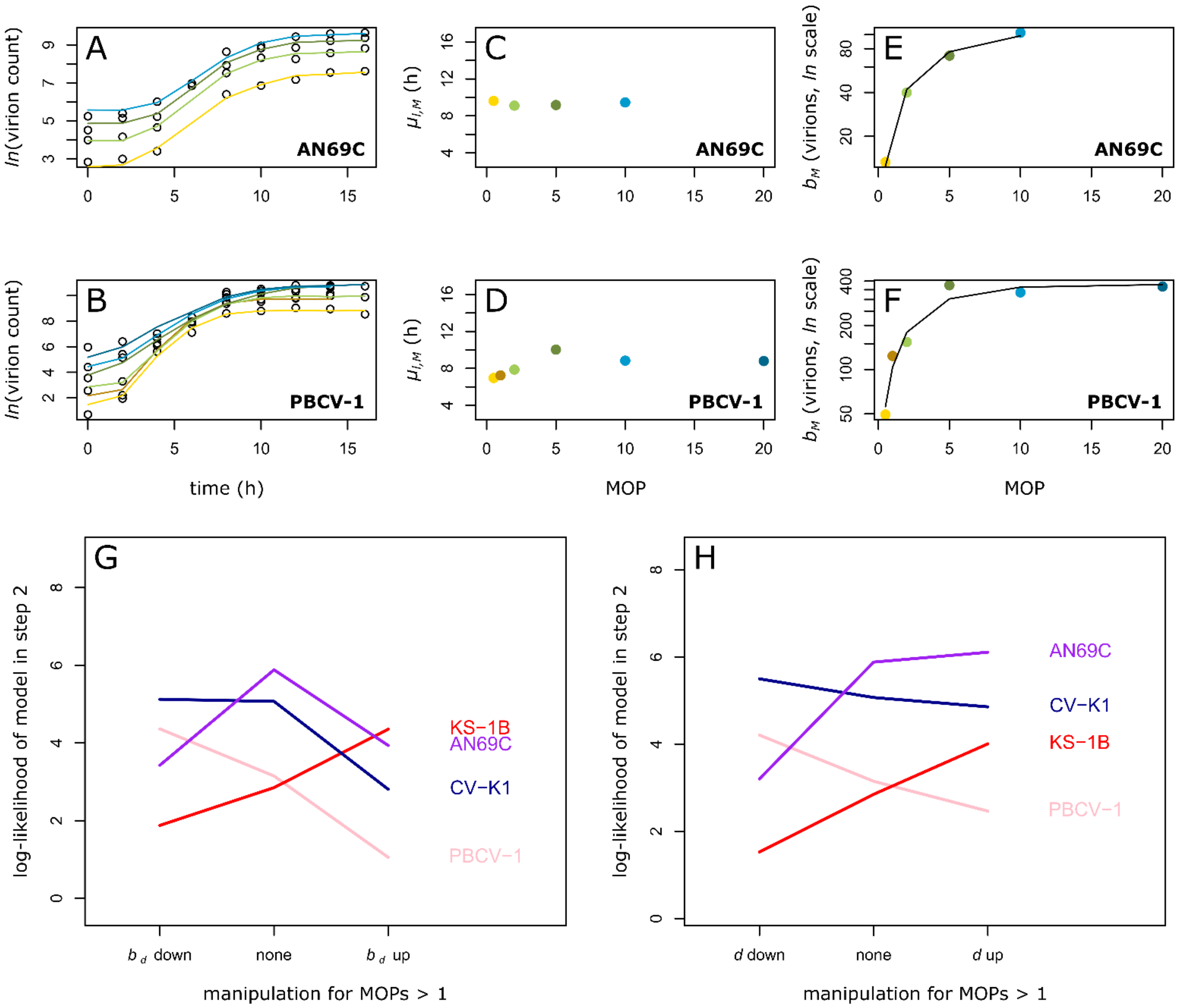

Supp. Fig. S6. Exploration of the assumption that mOSG parameters are independent of MOP. In our mOSG analysis models (Eq. 1), we assumed that all parameters were independent of MOP. This assumption enabled the separation of d and b_d, since it means that the amplitude of mOSG curves increases with MOP in a predictable and non-linear fashion. We evaluated whether our data supported this assumption in three steps (steps 1 & 2 following Lievens et al. 2022).

1) For every virus strain whose estimates of d, μ_l, and b_d were retained, we fit a model that did not assume independent depolarization probability, lysis time, or burst size: $V(t) = \frac{1}{\delta} * \left(A_a * M * e^{-k*A_a*t_a} + A_a * F\left(t, \mu_{l,M}, \sigma_{l,M}^2, \alpha\right) * b_M\right)$. This model fit a separate lysis time ($\mu_{l,M}$, $\sigma_{l,M}$) and average burst size per cell (b_M) to each MOP curve, but a joint adsorption constant k. The truncation value of lysis time (α) was set to 0. Examples are shown in **panels A-B** (open points: data, colored lines: model fits). The statistical fitting was done as for Eq. 1.

2) Based on the estimates from step 1 (colored points in **panels C-F**), we evaluated whether the assumption of independence was supported. For lysis time, we looked for trends in the $\mu_{l,M}$ estimates (examples in **panels C-D**). There were no visible trends, indicating that lysis time is independent of MOP. For depolarization probability and burst size, we investigated whether the estimates of b_M (i.e. the amplitude of the mOSG curves) increased as expected if MOP had no effect. We expected $b_M(M) = \left(1 - e^{-d*\left(1-e^{-k*A_a*t_a}\right)*M}\right) * b_d$, which is the probability that a cell is depolarized (a Poisson probability function of d and the MOA) multiplied by the average burst size per depolarized cell. We fit this model to the *ln*-transformed b_M fits using nonlinear least squares fitting ('nls' in base R, R Core Team 2014), then compared the b_M fits to the predicted curve (examples in **panels E-F**; black line: model fit for $b_M(M)$). We generally found a close correspondence between the two, suggesting that depolarization probability and burst size per depolarized cell are independent of MOP.

3) We investigated how sensitive step 2 was to deviations from independence for depolarization probability and burst size per depolarized cell. Note that this investigation was a proof of concept rather than a thorough power analysis. First, we generated four sets of artificial mOSG assay data. We predicted data using the strain's estimated traits and Eq. 1, but at MOPs > 1 set *i*) b_d to be 1.5 times higher, *ii*) b_d to be 1.5 times lower, *iii*) d to be 1.5 times higher, *iv*) d to be 1.5 times lower. This manipulated the (relative) amplitudes of the mOSG curves. We added realistic residuals, namely the residuals of the true data. We then ran steps (1) and (2) on the artificial datasets and compared their log-likelihood (i.e. their fit) with that of the true data. **Panels G and H** show the results of these comparisons for each strain (purple: AN69C, blue: CV-K1, red: KS-1B, pink: PBCV-1). In many cases, there is no clear difference between the fit of the Poisson probability model for the true and the manipulated data. This leads us to conclude that while our data does not conflict with the assumption of constant burst size, we have limited power to detect deviations from it.

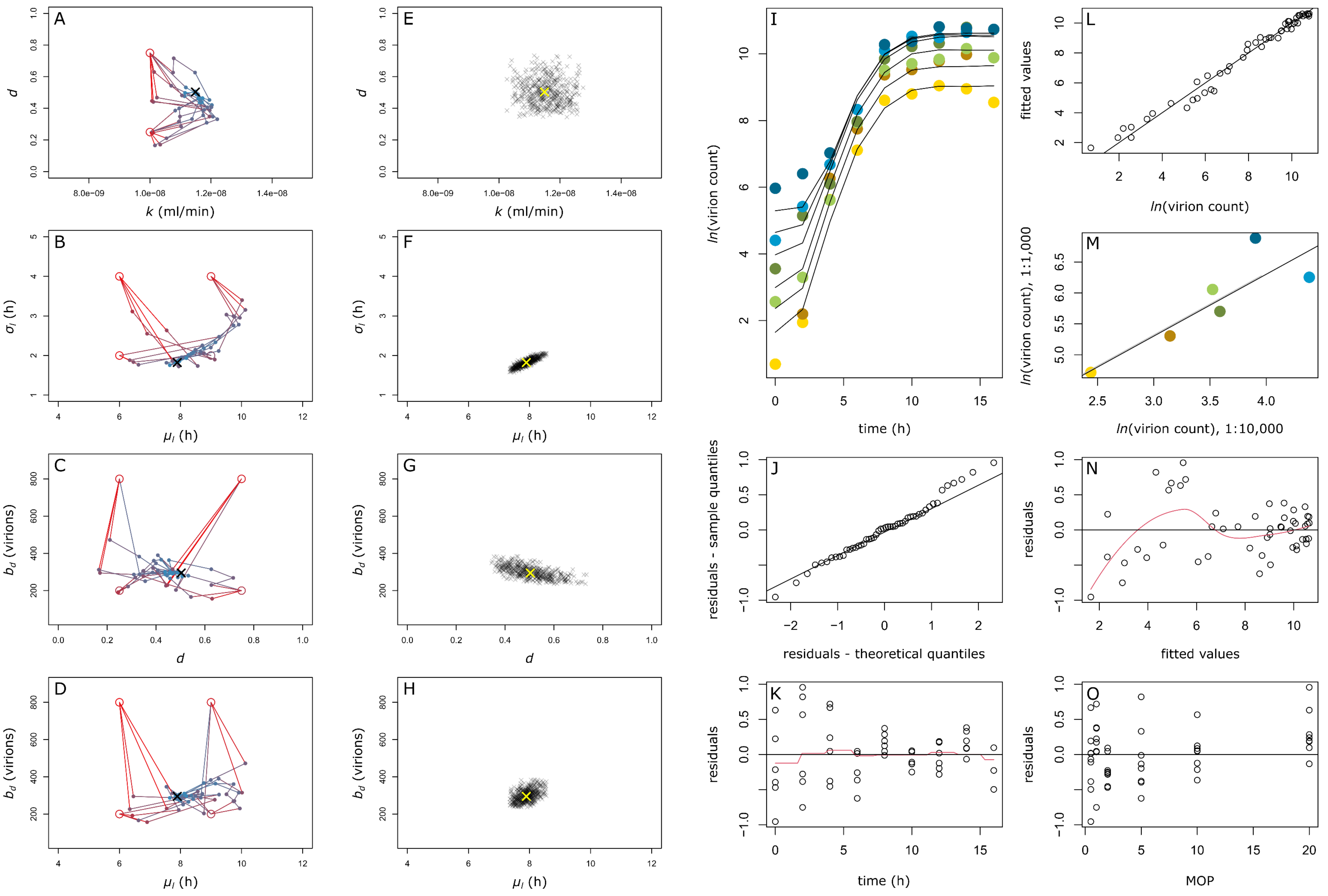

Supp. Fig. S7. Example of nonlinear least squares fitting for the mOSG model. The fitting is shown for strain PBCV-1. **A-D)** Traces of the model fitting process. For every viral strain, Eq. 1 was fit with 16 different combinations of initial parameters (open circles). Each fit converged to the estimated parameters (cross) through an iterative process (lines and solid points, colored red if the residual sum of squares was high and blue if it was low). Each panel represents this process for a different combination of parameters. **E-H)** Visualization of the bootstrapped confidence intervals around the estimated parameters (cross, here colored yellow for visibility). Bootstrapping was done by resampling residuals and recalculating Eq. 1 500 times; the 2.5 % and 97.5 % quantiles of the resulting estimates were taken as the 95 % CIs. The bootstrapped estimates that fell within the CI are shown here, for the same parameter combinations as in panels A-D. **I-O)** Assessment of model fit. **I&L)** Observed vs. predicted values. In panel I, points show the curated data (yellow: MOP 0.5, orange: 1, light green: 2, dark green: 5, blue: 10, indigo: 20) and lines the model fit. **M)** Comparison of the virion concentrations at 16 h for the 1 : 1,000 and 1 : 10,000 dilutions (data points colored as in panel I; the black line is the expectation if released virions do not start secondary infections). **J,K,N,O)** Independence and distribution of residuals.

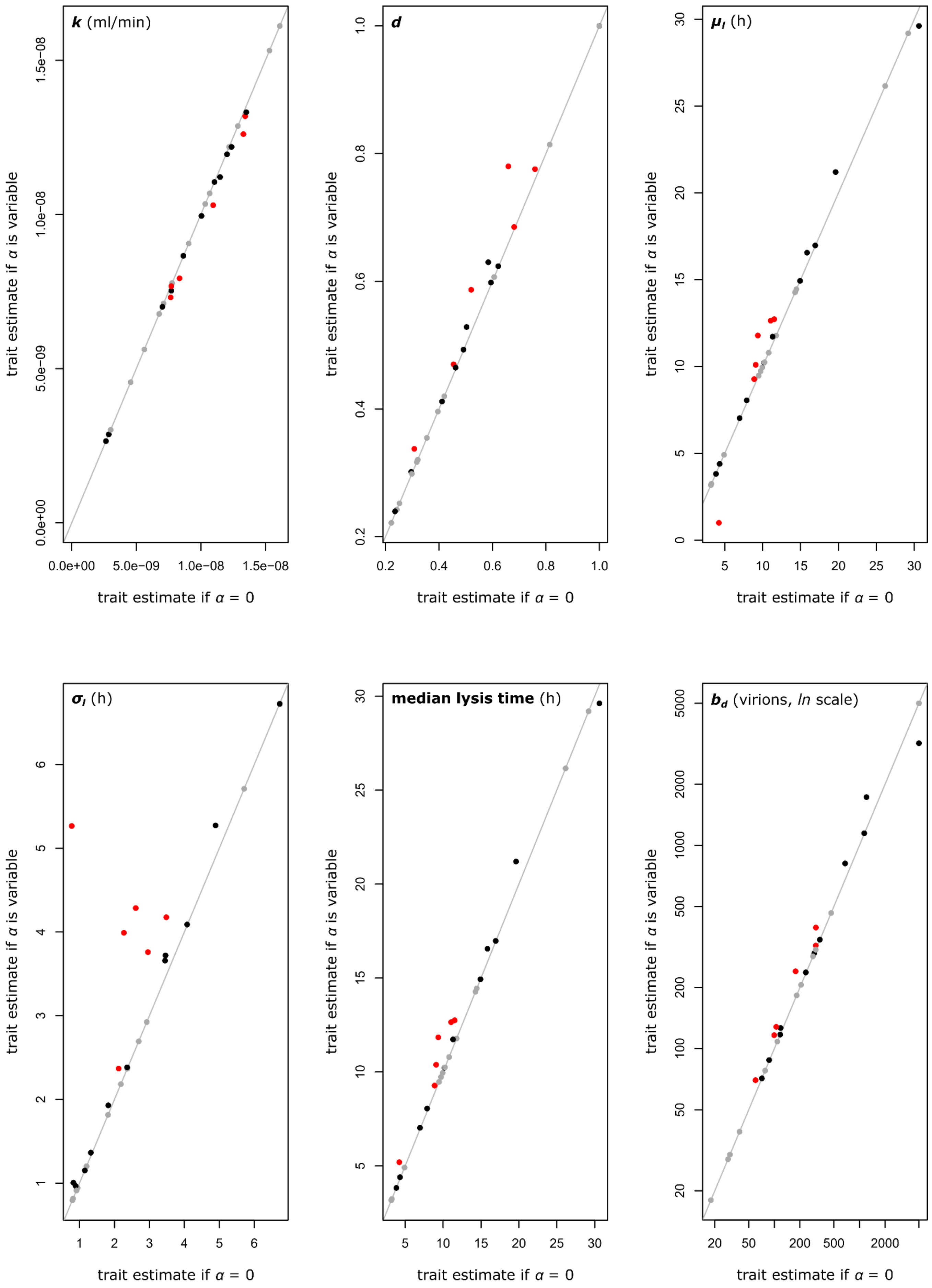

Supp. Fig. S8. Effect of imposing α = 0 in the mOSG model. For optimal illustration of the effects, this figure includes information from a larger chlorovirus dataset that will be published separately. Parameter α in Eq. 1 is the earliest time at which progeny virions can be produced. To investigate its effect, we fit Eq. 1 with α set to 0 or allowed to vary. For a minority of viral strains, the models with a variable α fit the data better based on the Akaike Information Criterion (ΔAIC > 2, Akaike 1974; red points); for the remainder the fit was equivocal (0 < ΔAIC < 2; black and gray points) and in many cases the estimated α value was 0 (gray points). This plot shows that setting α to 0 generally had a negligible effect on the other parameter estimates. Because the truncated normal distribution can be skewed, the estimated median lysis time (calculated based on μ_l, σ_l, and α) is also included. Lines: 1 : 1 line; points: trait estimates for one viral strain.

A.

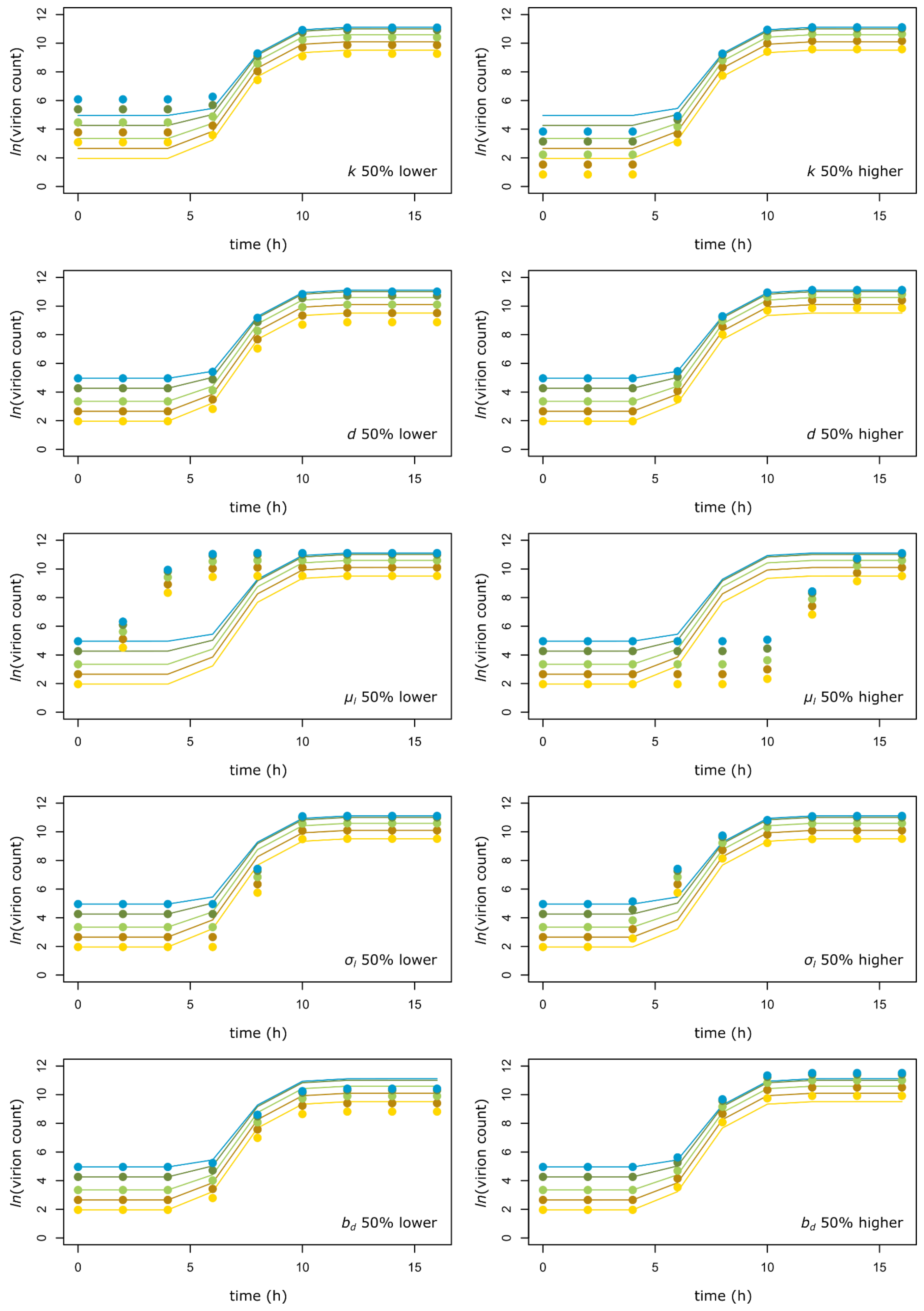

B.

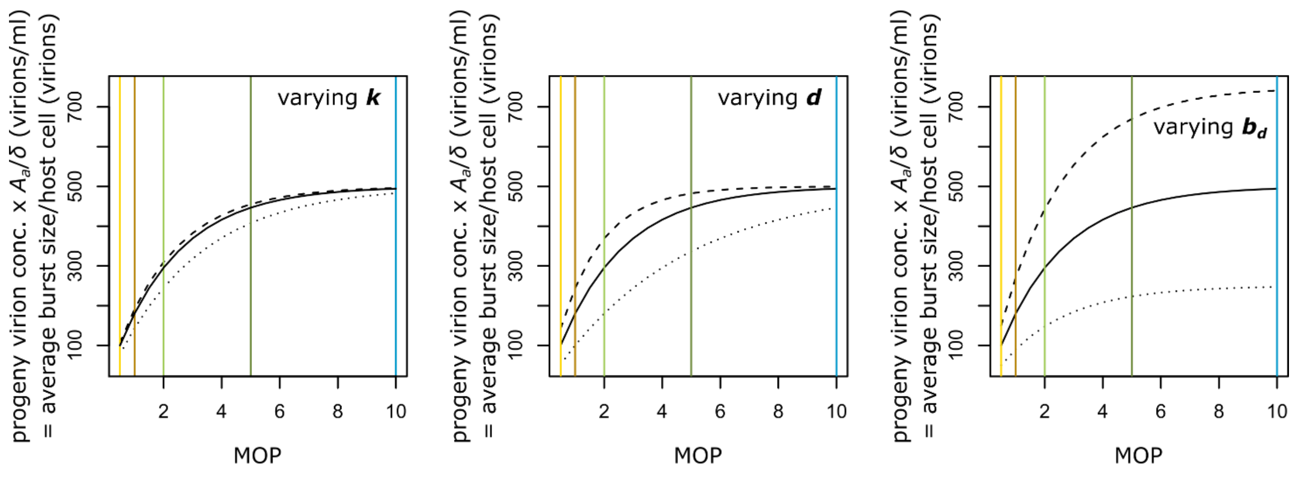

Supp. Fig. S9. Sensitivity of the mOSG model to its parameters. Panel A demonstrates each parameter's role in the mOSG model (Eq. 1). Lines represent a 'baseline' dataset, predicted for time points 0 h, 2 h, …, and 16 h and MOPs 0.5, 1, 2, 5, and 10 using Eq. 1. The baseline parameterization was: adsorption constant $k = 1 \times 10^{-8}$ ml/min, depolarization probability $d = 0.5$, mean lysis time $\mu_l = 9$ h, SD of lysis time $\sigma_l = 1$ h, burst size per depolarized cell $b_d = 500$ virions. For each row, we then decreased (left) or increased (right) one parameter by 50 % and plotted the resulting predictions as points. Colors indicate MOP: 0.5, yellow; 1, orange; 2, light green; 5, dark green; 10, blue. The effects of the mOSG parameters can be seen by comparing the baseline predictions (lines) to the varied predictions (points):

- *k*:
  - Diminished adsorption leads to higher concentrations of unadsorbed virions at the end of the adsorption period, i.e. to higher virion counts at time points 0-4 h. The reverse is true for improved adsorption.
  - For a given MOP, diminished adsorption leads to fewer infected cells, and thus to lower virion counts after the infected cells have lysed (time points 12-16 h). The reverse is true for improved adsorption. The impact of *k* is smaller at high MOPs (see panel B). Visually, variation in *k* has a small effect on the spacing between growth curves. *Note that this effect is minor compared to the effect of* k *at time points 0-4 h.*
- *d*: For a given MOP, lower depolarization probabilities lead to fewer infected cells, and thus to lower virion counts after the infected cells have lysed (time points 12-16 h). The reverse is true for higher depolarization probabilities. This effect is smaller at high MOPs (see panel B). Visually, variation in *d* affects the spacing between growth curves. *Note the difference with parameter* k*: here there is no effect on the virion counts at time points 0-4 h.*
- μ_l: Earlier mean lysis times cause earlier increases in virion counts. The reverse is true for later mean lysis times.
- σ_l: Less variation in lysis timing causes shallower increases in virion counts; more variation in lysis timing causes steeper increases in virion counts.
- b_d: Lower burst sizes lead to lower virion counts once the infected cells have lysed (time points 12-16 h). The reverse is true for higher burst sizes. This effect is not dependent on MOP (see panel B). Visually, variation in b_d affects the amplitude of the growth curves. *Note the difference with parameter* d*: here the highest possible virion count (at MOP 10) varies.*

Panel B explains why the effects of *k* and *d* are dependent on MOP, while b_d is not. MOP has a saturating effect on the concentration of infected cells: increasing MOP increases the concentration of infected cells, but only until 100 % of the cells are infected. Since the concentration of infected cells determines the concentration of progeny virions once lysis has occurred, MOP also has a saturating effect on the concentration of progeny virions. The concentration of progeny virions once lysis has occurred is $\frac{A_a}{\delta} * \left(1 - e^{-d*(1-e^{-k*A_a*t_a})*M}\right) * b_d$ (Eq. 1, second term with $F(t; \mu_l, \sigma_l^2, \alpha)$ set to 1). This is equivalent to the concentration of all algal cells ($\frac{A_a}{\delta}$) multiplied by the average burst size of all algal cells (including cells without attached virions and cells that were not depolarized). We plotted this curve for MOPs 0.5-10, using the same baseline parameterization as in panel A (solid lines). Dotted and dashed lines represent respectively 50 % lower and 50 % higher parameter values of *k* (left), *d* (middle), and b_d (right). While variation in *k* and *d* affects the slope, variation in b_d affects the saturation point. Horizontal lines indicate the MOPs used in panel A.

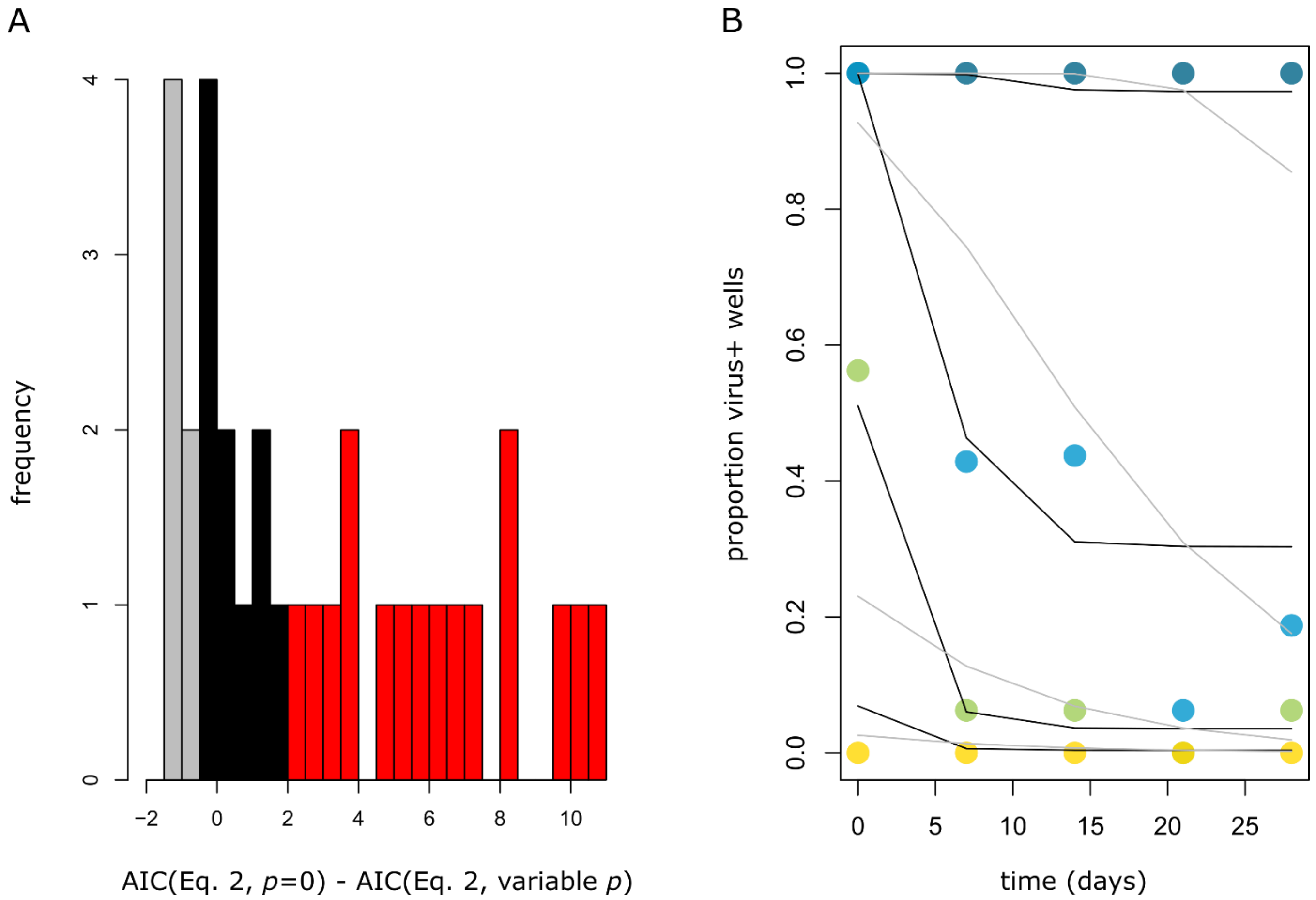

Supp. Fig. S10. Effect of imposing p = 0 in the mS model. For optimal illustration of the effects, this figure includes information from a larger chlorovirus dataset that will be published separately. Parameter p in Eq. 2 is the proportion of initially infectious virions that are robust to decay. To investigate its effect, we fit Eq. 2 with p set to 0 or allowed to vary. **A)** For half of the viral strains, the models with a variable p fit the data better based on the Akaike Information Criterion (ΔAIC > 2, Akaike 1974; red bars). In cases where the fit was equivocal (0 < ΔAIC < 2; black and gray bars), the estimated p was often 0 or 1 (gray bars). **B)** Example of a case where variable p fit the data better. Shown are the observed vs. predicted values for chlorovirus strain NE-JV-4: the black line shows the predicted values when p is variable (AIC 20.8), the gray line shows the predicted values when p is set to 0 (AIC 28.9). Note that the gray lines underestimate the proportion of virus-positive wells at t = 0.

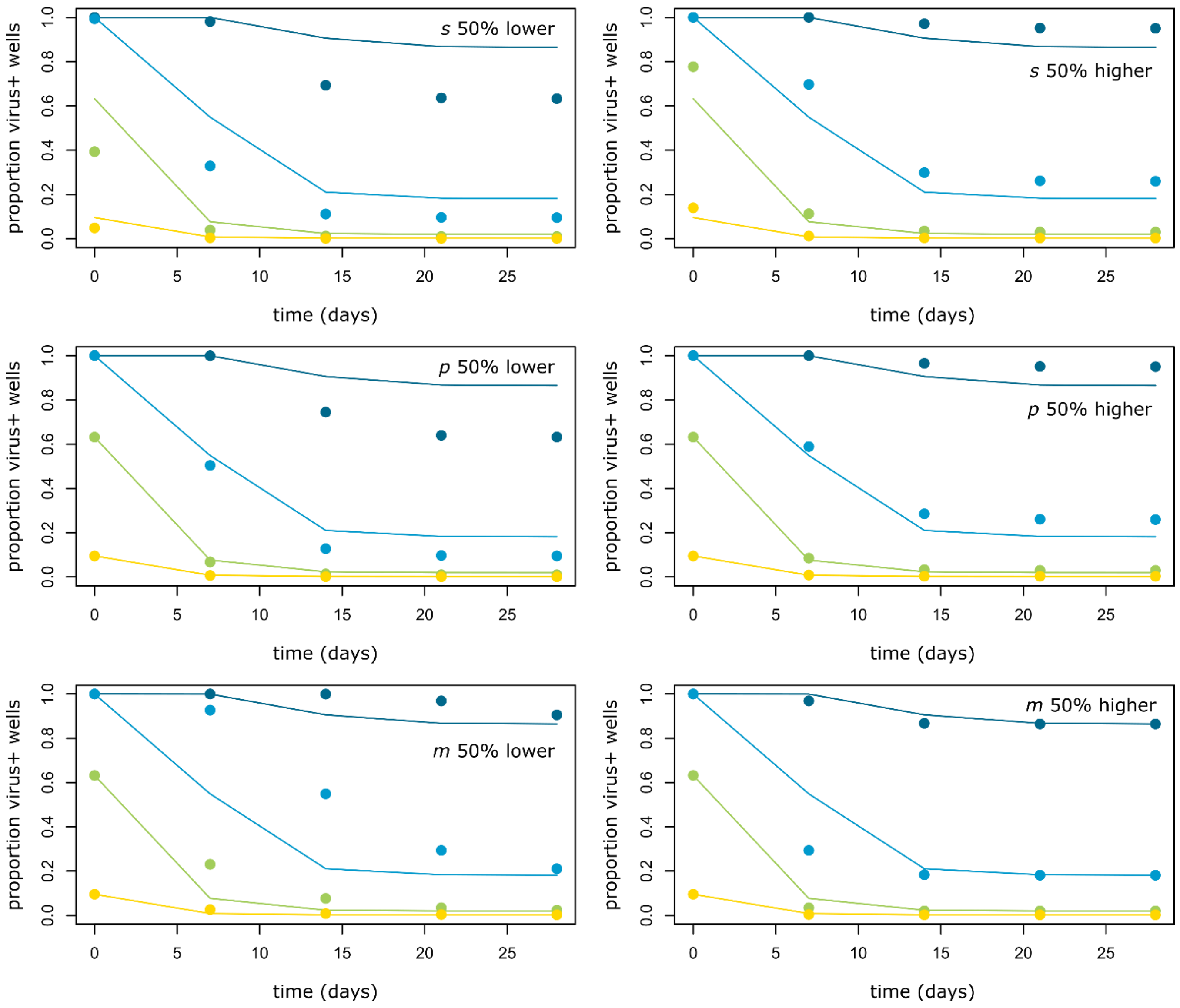

Supp. Fig. S11. Sensitivity of the mS model to its parameters. Here we demonstrate each parameter's role in the mS model (Eq. 2). Lines represent a 'baseline' dataset, predicted for time points 0 h, 7 h, …, and 28 d and initial virion numbers 0.5, 5, 50, and 500 using Eq. 2. The baseline parameterization was: specific infectivity s = 0.2, persistent fraction p = 0.02, mortality rate m = 0.5. For each row, we then decreased (left) or increased (right) one parameter by 50 % and plotted the resulting predictions as points. Colors indicate the number of initial virions added: 0.5, yellow; 5, light green; 50, blue; 500, indigo. The effects of the mS parameters can be seen by comparing the baseline predictions (lines) to the varied predictions (points):

- *s:* Lower specific infectivity leads to a lower proportion of virus-positive wells throughout the entire assay, most noticeably at time point 0. The reverse is true for higher specific infectivity. *Note the difference with parameters* p *and* m*, which do not affect the proportion of virus-positive wells at time point 0.*
- *p*: Variation in the persistent fraction is detectable at later time points, when the nonpersistent virions have decayed (time points 21-28 days). Smaller persistent fractions mean the proportion of virus-positive wells converges to a lower number; the reverse is true for larger persistent fractions.
- *m*: Variation in mortality rate is detectable at intermediate time points (7-14 days). Lower mortality rates slow the decline in virus-positive wells, i.e. slow the convergence towards the persistent fraction. The reverse is true for higher mortality rates.

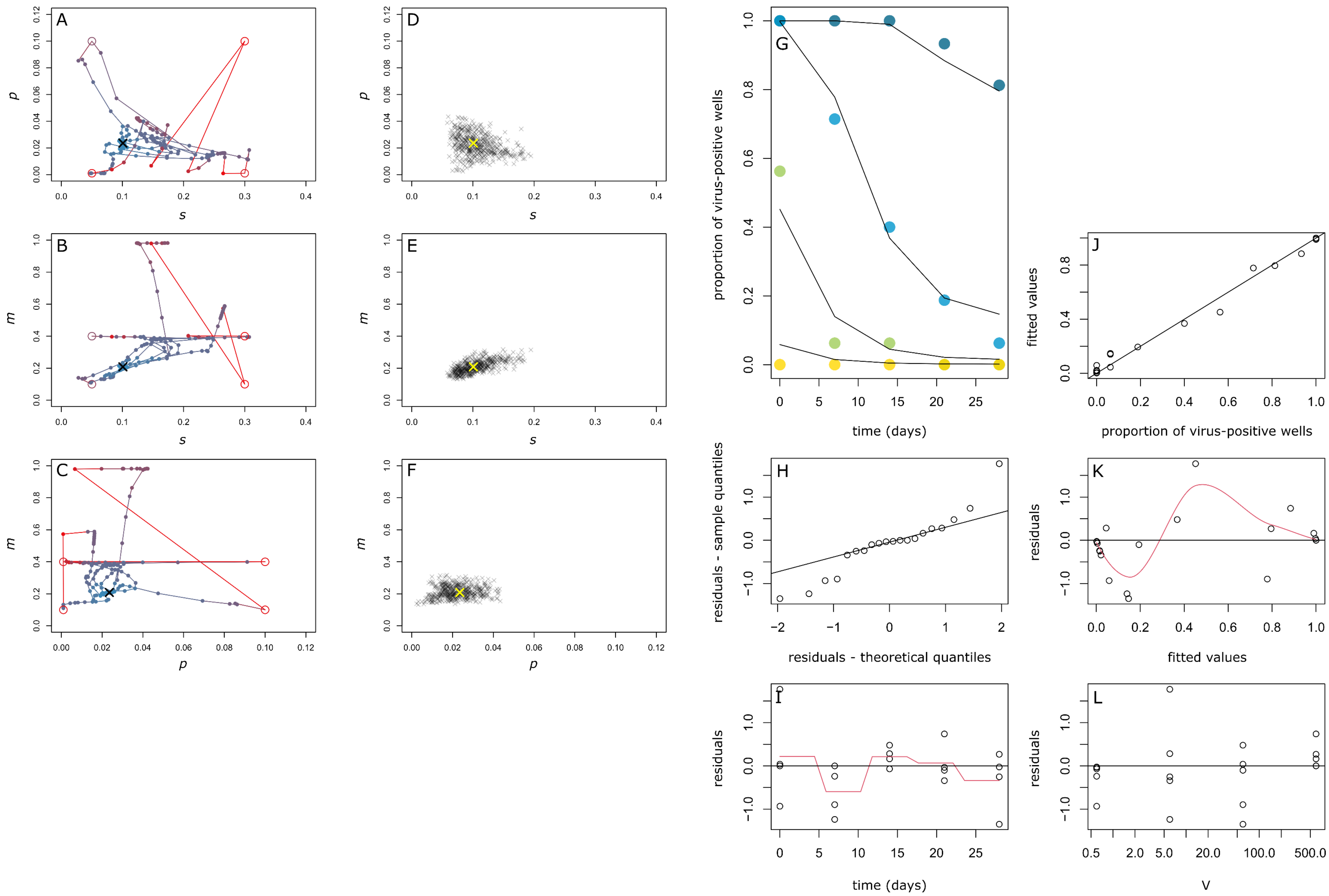

Supp. Fig. S12. Example of generalized nonlinear regression fitting for the mS model. The fitting is shown for strain PBCV-1. **A-C)** Traces of the model fitting process. For every viral strain, Eq. 2 was fit with 8 different combinations of initial parameters (open circles). Each fit converged to the estimated parameters (cross) through an iterative process (lines and solid points, colored red if the minimizing function was high and blue if it was low). Each panel represents this process for a different combination of parameters. **D-F)** Visualization of the bootstrapped confidence intervals around the estimated parameters (cross, here colored yellow for visibility). Bootstrapping was done by simulating data from the estimated parameters and recalculating Eq. 2. 500 times; the 2.5 % and 97.5 % quantiles of the resulting estimates were taken as the 95 % CIs. The bootstrapped estimates that fell within the CI are shown here, for the same parameter combinations as in panels A-C. **G-L)** Assessment of model fit. **G&J)** Observed vs. predicted values. In panel G, points show the curated data (yellow: 0.5 initial virions added, light green: 5, blue: 50, indigo: 500) and lines the model fit. **H,I,K,L)** Independence and distribution of residuals (we do not expect the residuals to be normally distributed here, as the data is binomial with limited replication).
