# Supplementary material for: Efficient assays to quantify the life history traits of algal viruses
Source: Appl Environ Microbiol. 2023 Nov 21;89(12):e01659-23. doi: 10.1128/aem.01659-23 (PMC10734466; doi:10.1128/aem.01659-23)
Supplement: Supplemental file 2 — Supplemental methods. [file aem.01659-23-s0002.pdf]

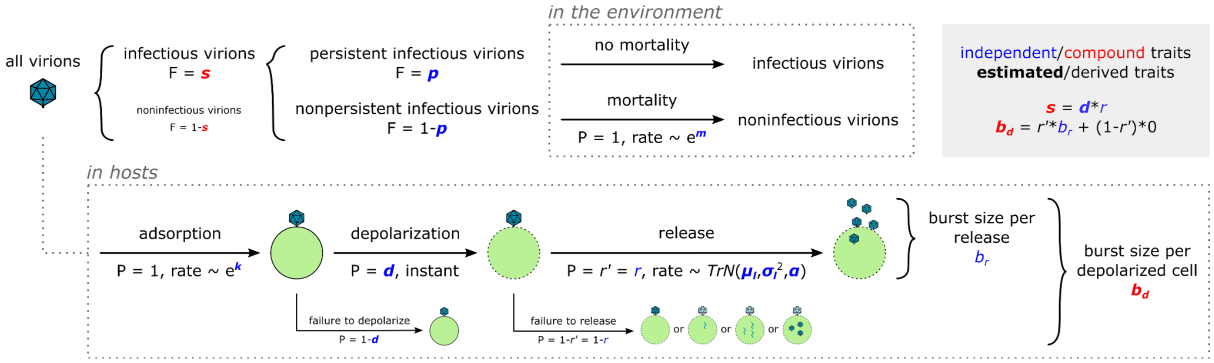

Figure M1. Life cycle of the chloroviruses, as assumed in our statistical models. See the text and Table 1 for definitions of the traits. Traits marked in blue describe independent life cycle steps; traits marked in red arise from combinations of two or more steps (gray box). Traits in bold are estimated from the modified one-step growth (mOSG) or modified survival (mS) assays; traits not in bold are derived by comparing the two assays. Note that in this section, we distinguish the probability *r* (the probability that a depolarized cell releases infectious virions) from *r′* (the probability that a depolarized cell releases virions, infectious or not). This is done for completeness, but we assume the two probabilities are equal. Abbreviations: F, initial frequency; P, probability; *TrN*, truncated normal distribution.

Derivation of Eq. 1 (mOSG assay)

The free virion concentration at each time point $V(t)$ was made up of unadsorbed virions and newly released progeny virions, i.e.

$$V(t) = \frac{V_0}{\delta} + V_p \qquad \text{(Eq. S1.1)},$$

where δ is the dilution factor, V_0 is the concentration of unadsorbed virions at the end of the adsorption period, and V_p is the concentration of progeny virions in the diluted suspension. V_p is determined by the concentration of depolarized algae at the end of the adsorption period and after dilution A_d/δ, the probability that a depolarized cell releases virions r', the proportion of cells that released virions over time *F(t)*, and the average number of progeny virions per release b_r (Fig. M1):

$$V(t) = \frac{V_0}{\delta} + \frac{A_d}{\delta} * r' * F(t) * b_r \qquad \text{(Eq. S1.2)}.$$

$r'*b_r$ reduces to b_d, the burst size per depolarized cell (see Fig. M1), so

$$V(t) = \frac{1}{\delta} * (V_0 + A_d * F(t) * b_d) \qquad \text{(Eq. S1.3)}.$$

V_0, A_d, and *F(t)* are described in more detail below. Note that we assume $b_{r'}$ and b_d are constant, and not contingent on MOP. This assumption is based on the finding that chloroviruses mutually exclude each other through depolarization (Greiner et al. 2009). Therefore we can assume that all depolarized cells behave like cells infected by one virion, no matter how many virions capable of

depolarization virions are attached to them. This is supported by measurements of b_r in single cells (Lievens et al. 2022), at least in the range of MOPs we use (very high MOIs can be associated with differences and dual infections, Van Etten et al. 1983, Tessman 1985, Chase et al. 1989).

V_0 is the concentration of unadsorbed virions at the end of the adsorption period (i.e. at t = 0, right before dilution). We assume that there are enough binding sites available for all virions (Meints et al. 1988), that all virions are capable of adsorbing, and that virions adsorb at a constant rate represented by the adsorption constant k. The concentration of unadsorbed virions is then

$$V_0 = A_a * M * e^{-k*A_a*t_a} \quad \text{(Eq. S2)}$$

(Hyman and Abedon 2009, eq. 18.2), where A_a is the algal concentration during the adsorption period, M is the MOP, A_a*M is the free virion concentration at the start of the adsorption period, and t_a is the duration of the adsorption period in minutes.

A_d is the concentration of depolarized algae at the end of the adsorption period. We assume that adsorbed virions are randomly distributed across host cells, and that virions capable of depolarizing host cells adsorb at the same rate as virions incapable of depolarizing host cells. In that case, the average number of virions capable of depolarization that adsorb to each cell is $d*M_A$, where d is the proportion of virions capable of depolarizing a host cell (i.e. the depolarization probability when a virion is adsorbed) and M_A is the multiplicity of adsorption (*sensu* Hyman and Abedon 2009). The concentration of algae with at least one depolarization-capable virion attached can then be calculated using the Poisson distribution with expected number of events $d*M_A$:

$$A_d = A_a * \left(1 - e^{-d*M_A}\right) \quad \text{(Eq. S3.1).}$$

The multiplicity of adsorption is the concentration of adsorbed virions (the initial virion concentration minus the unadsorbed virion concentration) divided by the concentration of algal cells:

$$M_A = \frac{M*A_a - V_0}{A_a} = \left(1 - e^{-k*A_a*t_a}\right) * M \quad \text{(Eq. S4)}$$

(see Eq. S2). Thus the concentration of depolarized algae is

$$A_d = A_a * \left(1 - e^{-d*\left(1 - e^{-k*A_a*t_a}\right)*M}\right) \quad \text{(Eq. S3.2).}$$

This non-linear function reflects the fact that maximum 100% of the algae can be depolarized. For example, when $d*M_A$ is 0.5, the fraction of depolarized algae is expected to be 39% (Eq. S3.1). Doubling the MOP (and thus doubling $d*M_A$, Eq. S4) would increase that fraction to 63%. When the initial $d*M_A$ is 5, however, the fraction of depolarized algae is already 99% and doubling it would have little effect. When combined with the constant burst size (see above), this is the key insight that

allows us to disentangle burst size from depolarization probability: b_d determines the concentration of progeny virions at saturating MOPs, while *d* determines the saturation point (Supp. Fig. S10).

F(t), the proportion of lysed cells over time, deviates from an idealized 'step' pattern due to variation among the host cells (Rabinovitch et al. 1999). Determining the precise distribution of lysis time across the host population is not trivial (e.g. Adams and Wassermann 1956, Dennehy and Wang 2011), but we have found the truncated normal distribution to be an acceptable approximation given the simplification that all depolarized host cells produce b_d virions (methods as in Adams and Wassermann 1956; data not shown). Thus *F(t)* follows the cumulative distribution function of a truncated normal distribution:

$$F\left(t;\, \mu_l,\, \sigma_l^2,\, \alpha\right) \qquad \text{(Eq. S5).}$$

This function expresses the proportion of lysed cells at *t* hours after dilution, given a mean lysis time μ_l, standard deviation σ_l, and earliest possible lysis time α.

Plugging Eqs. S2, 3.2, and 5 back into Eq. S1.3, the concentration of virions in the diluted suspensions is:

$$V(t) = \frac{1}{\delta} * \left(A_a * M * e^{-k*A_a*t_a} + A_a * \left(1 - e^{-d*\left(1-e^{-k*A_a*t_a}\right)*M}\right) * F\left(t;\, \mu_l,\, \sigma_l^2,\, \alpha\right) * b_d\right) \qquad \text{(Eq. 1).}$$

In this study, we set the lysis time truncation value α to 0 for simplicity (including α as a separate parameter had minimal effects on the parameter estimates, Supp. Fig. S7).

Derivation of Eq. 2 (mS assay)

The proportion of virus-positive wells over time follows a binomial distribution with 16 trials and a success probability of *P(t)*. The success probability is the probability that at least one infectious virion was added to a well. It can be calculated using the Poisson distribution with expected number of events *λ(t)*:

$$P(t) = 1 - e^{-\lambda(t)} \qquad \text{(Eq. S6).}$$

If mortality follows the exponential decay model (following e.g. Cottrell and Suttle 1995, Noble and Fuhrman 1997, Demory et al. 2021), the expected number of infectious virions per well is

$$\lambda(t) = s * V * e^{-m*t} \qquad \text{(Eq. S7).}$$

Here the mortality term is e^{-m*t}, where *m* is the constant mortality rate and *t* is the time in days. The number of infectious virions at *t = 0* is given by the initial number of virions *V* (10 µl of 5×10^4,

5×10^3, 5×10^2, and 5×10^1 virions/ml suspensions, i.e. 500, 50, 5, or 0.5 virions) and the initial proportion of infectious virions *s*.

However, in this and other work we found that Eq. S7 was a poor fit for many virus strains (Supp. Fig. S8). Upon investigation, these strains had higher mortality at the beginning of the assay than at the end. Therefore, we added an additional parameter *p*:

$$\lambda(t) = V * s * p + V * s * (1 - p) * e^{-m*t} \quad \text{(Eq. S8).}$$

This is a biphasic model, where the 'expected number of infectious virions' term is split into two parts: $s * p * V$ represents a subpopulation of persistent infectious virions, which do not decay over time; $s * (1 - p) * V * e^{-m*t}$ represents a subpopulation of nonpersistent infectious virions, which decay exponentially. The probability that an infectious virion is persistent is *p*. This parametrization is the simplest extension of Eq. S7, and fit our data very well. We don't exclude that other possibilities, e.g. a model where the persistent fraction decays at a slower rate, could also fit the data well.

Plugging Eq. S8 into Eq. S6, the expected proportion of virus-positive wells is:

$$P(t) = 1 - e^{-(V*s*p+V*s*(1-p)*e^{-m*t})} \quad \text{(Eq. 2).}$$

Derivation of Eqs. 3 & 4 (Comparison of mOSG and mS assays)

The probability that a virion is infections, i.e. that it can complete life cycle steps 1-3 (Fig. 1), is the product of three probabilities: the probability that a virion is capable of adsorption (step 1; assumed to be 1), the probability that an adsorbed virion is capable of depolarization *d* (step 2), and the probability that a depolarized cell is capable of releasing infectious virions *r* (step 3). The specific infectivity *s* describes the initial proportion of infectious virions in the mS assay, which is equivalent to the probability that any given virion is infectious. Thus

$$s = 1 * d * r \quad \text{(Eq. S9),}$$

which produces Eq. 3.

The burst size per release of virions b_r (see Eq. S1.2) can be calculated from the probability that a depolarized cell releases virions *r'* and the burst size per depolarized cell b_d, since

$$b_d = r' * b_r + (1 - r') * 0 \quad \text{(Eq. S10.1)}$$

$$b_d = r' * b_r \quad \text{(Eq. S10.2)}$$

(Fig. M1). Assuming that all bursts contain a mix of infectious and noninfectious virions, the probability that a depolarized cell releases virions *r'* is equal to the probability that a depolarized cell releases infectious virions *r*. Therefore

$$b_d = r * b_r \quad \text{(Eq. S10.3),}$$

which produces Eq. 4.

References

Adams, M. H., and F. E. Wassermann. 1956. Frequency Distribution of Phage Release in the One-Step Growth Experiment. Virology 2:96–108.

Chase, T. E., J. A. Nelson, D. E. Burbank, and J. L. Van Etten. 1989. Mutual Exclusion Occurs in a *Chlorella*-like Green Alga Inoculated with Two Viruses. Journal of General Virology 70:1829–1836.

Cottrell, M. T., and C. A. Suttle. 1995. Dynamics of a lytic virus infecting the photosynthetic marine picoflagellate *Micromonas pusilla*. Limnology and Oceanography 40:730–739.

Demory, D., J. S. Weitz, A.-C. Baudoux, S. Touzeau, N. Simon, S. Rabouille, A. Sciandra, and O. Bernard. 2021. A thermal trade-off between viral production and degradation drives virus-phytoplankton population dynamics. Ecology Letters 24:1133–1144.

Dennehy, J. J., and I.-N. Wang. 2011. Factors influencing lysis time stochasticity in bacteriophage l. BMC Microbiology 11:174.

Greiner, T., F. Frohns, M. Kang, J. L. Van Etten, A. Käsmann, A. Moroni, B. Hertel, and G. Thiel. 2009. Chlorella viruses prevent multiple infections by depolarizing the host membrane. Journal of General Virology 90:2033–2039.

Hyman, P., and S. T. Abedon. 2009. Practical Methods for Determining Phage Growth Parameters. Pages 175–202 *in* M. R. J. Clokie and A. M. Kropinski, editors. Bacteriophages: Methods and Protocols, Volume 1: Isolation, Characterization, and Interactions, vol. 401. Humana Press.

Lievens, E. J. P., M. Spagnuolo, T. Réveillon, and L. Becks. 2022. Delayed lysis time at high multiplicities of particles in a chlorovirus-*Chlorella* interaction. Microbes and Environments 37:ME22068.

Meints, R. H., D. E. Burbank, J. L. Van Etten, and D. T. A. Lamport. 1988. Properties of the chlorella receptor for the virus PBCV-1. Virology 164:15–21.

Noble, R. T., and J. A. Fuhrman. 1997. Virus decay and its causes in coastal waters. Applied and Environmental Microbiology 63:77–83.

Rabinovitch, A., H. Hadas, M. Einav, Z. Melamed, and A. Zaritsky. 1999. Model for Bacteriophage T4 Development in *Escherichia coli*. Journal of Bacteriology 181:1677–1683.

Tessman, I. 1985. Genetic recombination of the DNA plant virus PBCV1 in a *Chlorella*-like alga. Virology 145:319–322.

Van Etten, J. L., D. E. Burbank, Y. Xia, and R. H. Meints. 1983. Growth cycle of a virus, PBCV-1, that infects *Chlorella*-like algae. Virology 126:117–125.
